# Functional Imaging of Microbial Interactions With Tree Roots Using a Microfluidics Setup

**DOI:** 10.3389/fpls.2020.00408

**Published:** 2020-04-15

**Authors:** Marie-Francoise Noirot-Gros, Shalaka V. Shinde, Chase Akins, Jessica L. Johnson, Sarah Zerbs, Rosemarie Wilton, Kenneth M. Kemner, Philippe Noirot, Gyorgy Babnigg

**Affiliations:** Biosciences Division, Argonne National Laboratory, Lemont, IL, United States

**Keywords:** root-microbe interaction, *Populus tremuloides*, *Pseudomonas fluorescens*, *Bacillus subtilis*, biosensor, microfluidics

## Abstract

Coupling microfluidics with microscopy has emerged as a powerful approach to study at cellular resolution the dynamics in plant physiology and root-microbe interactions (RMIs). Most devices have been designed to study the model plant *Arabidopsis thaliana* at higher throughput than conventional methods. However, there is a need for microfluidic devices which enable *in vivo* studies of root development and RMIs in woody plants. Here, we developed the RMI-chip, a simple microfluidic setup in which *Populus tremuloides* (aspen tree) seedlings can grow for over a month, allowing continuous microscopic observation of interactions between live roots and rhizobacteria. We find that the colonization of growing aspen roots by *Pseudomonas fluorescens* in the RMI-chip involves dynamic biofilm formation and dispersal, in keeping with previous observations in a different experimental set-up. Also, we find that whole-cell biosensors based on the rhizobacterium *Bacillus subtilis* can be used to monitor compositional changes in the rhizosphere but that the application of these biosensors is limited by their efficiency at colonizing aspen roots and persisting. These results indicate that functional imaging of dynamic root-bacteria interactions in the RMI-chip requires careful matching between the host plant and the bacterial root colonizer.

## Introduction

The plant microbiome plays an important role in the rhizosphere (Compant et al., 2005; Brown et al., 2012; Bulgarelli et al., 2013). Virtually all plant tissues host microbes that can act as symbionts, commensals, or pathogens. Interactions between plant and microbes can be beneficial, neutral, or harmful and directly influence plant growth and productivity (Compant et al., 2005; Newton et al., 2010; Gaiero et al., 2013). Plant-growth-promoting (PGP) rhizobacteria are bacteria that exert beneficial effects on plants through direct or indirect interactions with the roots (Varivarn et al., 2013; Dupuy and Wendy, 2016). PGP bacteria have the potential to increase the availability of soil nutrients to the plant, produce metabolites such as plant hormones, elicit plant systemic defenses, and increase plant resistance to biotic and abiotic stresses (Rodriguez and Fraga, 1999; Barea et al., 2005). In return, the plant provides photosynthetically derived carbon, such as sugars and organic acids that are consumed by rhizosphere microorganisms as well as a wide range of molecular compounds acting as environmental signals for the root microbiota. Microbes attach to the root surface and form micro-colonies that can eventually grow into larger biofilms. The formation of biofilms at root surfaces was proposed to be part of the cellular PGP activities of beneficial rhizobacteria (Seneviratne et al., 2010).

Understanding the complex interactions between plant roots and microbes requires the ability to track their dynamics at high spatial and temporal resolution. Real-time monitoring of dynamic root-microbe interactions (RMIs) at cellular resolution is now possible using microfluidics approaches coupled with advanced live imaging microscopy. Microfluidic platforms provide a powerful approach to evaluate the responses of growing plant cells to external perturbations (e.g., nutrients, media flow, temperature, hydrodynamics, light, and stressors) at throughputs higher than with conventional methods using pots or plates, and in precisely controlled environments. Multiple microfluidics devices such as “Plant on a chip” (Meier et al., 2010), RootChip (Grossmann et al., 2011), RootArray (Busch et al., 2012), TipChip (Agudelo et al., 2013), and PlantChip (Jiang et al., 2014) were developed to study various aspects of the cell biology of *Arabidopsis thaliana*, including gene expression, cell biomechanics, cellular mechanism of growth and cell division (reviewed in Sanati Nezhad, 2014). The PlantChip device enables the continuous monitoring of phenotypic changes at the cellular level and also at the whole plant level, including seed germination and root and shoot growth (hypocotyls, cotyledons, and leaves) (Jiang et al., 2014).

Fewer studies have used microfluidic devices to visualize the interactions of Arabidopsis roots with pathogenic or beneficial microorganisms. Using the plant-in-chip platform, visualization of the attack of Arabidopsis roots by pathogenic nematodes and oomycetes motile spores revealed some physiological changes taking place in the host plant and the pathogen during the attack (Parashar and Pandey, 2011). Recently, a microfluidic device to track root interactions system (TRIS) revealed a distinct chemotactic behavior of the bacterium *Bacillus subtilis* toward the root elongation zone and its rapid colonization, and allowed real-time monitoring of bacterial preference between roots from various Arabidopsis genotypes (Massalha et al., 2017). Another recent study investigated the spatiotemporal dynamics of colonization of Arabidopsis roots by PGP bacterial species from the *P*opulus *deltoids* rhizosphere over 4 days (Aufrecht et al., 2019). To date, studies of plant roots and RMIs using microfluidics have been focused on *A. thaliana*, an annual herbaceous plant model that can complete is entire life cycle in 6 weeks and grows a single primary root that later produces smaller lateral roots. However, there is a need to study root development and RMIs for other plants, including woody perennial plants such as trees.

Here, we describe a microfluidic device, called RMI-chip, that enables the direct visualization of RMIs taking place at early stages of tree seedling growth. We studied the interactions of *Populus tremuloides* (trembling aspen tree) with the bacterium *Pseudomonas fluorescens.* These interactions are biologically relevant in nature, as *P. fluorescens* is abundant in the rhizosphere of *Populus* trees (Gottel et al., 2011; Brown et al., 2012; Weston et al., 2012; Timm et al., 2015), and exhibits functionality in laboratory assays. We have shown that *P. fluorescens* promotes the growth of aspen seedlings (Shinde et al., 2017), colonizes aspen seedling roots by forming dense and dynamic biofilms (Noirot-Gros et al., 2018), and modulates expression of anti-fungal defense response genes in roots of aspen seedlings (Shinde et al., 2019). The RMI-chip device was designed to accommodate aspen seedling growth for periods up to 5 weeks, and to enable direct observation of root growth and its dynamic colonization by *P. fluorescens* biofilms with high spatiotemporal resolution. The RMI-chip was also used to monitor the growth of rice seedling roots and detect the production of reactive oxygen species (ROS) by the root using engineered *B. subtilis* strains as biosensors. We find that in the RMI-chip interactions between host plants and bacterial species are specific, consistent with ecological observations and with colonization profiles observed in other experimental systems, and that formation of bacterial biofilms on root surfaces is needed for persistent colonization.

## Materials and Methods

### Seeds and Growth Media

Seeds of *P. tremuloides* Michx. were obtained from the National Tree Seed Center, Natural Resources Canada, Fredericton, NB, Canada. The rice seeds were obtained from Baker Creek Heirloom Seed Co. (Mansfield, MO, United States). Phytoblend was purchased from Caisson Laboratories, Inc. (Smithfield, UT, United States). All other chemicals were purchased from Sigma-Aldrich (St. Louis, MO, United States).

### RMI-Chip and Humidity Chamber Design

The chips were designed with AutoCAD software (Autodesk) and were fabricated via soft lithography at the Scientific Device Laboratory (Des Plaines, IL, United States). Briefly, SU-8 2025 photoresist (MicroChem, Westborough, MA, United States) was used to make molds on a 4-inch silicon wafer. The two-part silicone elastomer (SYLGARD 184, Thermo Fisher Scientific, Waltham, MA, United States), the silane precursor and curing agent were mixed 10:1, degassed, poured on silicon wafer mold, degassed and baked at 65°C for 4 h. The polydimethylsiloxane (PDMS) elastomer was punched and bonded to a 48 × 65mm, thickness No. 1 cover glass (Ted Pella Inc., Redding, CA, United States). The humidity chambers and microscope stage adapters were fabricated from PMMA thermoplastic via laser cutting (Ponoko, Oakland, CA, United States) and assembled with acrylic adhesive (Weld-On, Compton, CA, United States). Tubing management and other holder accessories were 3D printed using PLA. The AutoCAD files (ESI) of the RMI-chip design, the SVG files used for laser cutting, and the SCAD and STL files used for 3D printing are available in Supplementary Material.

### Construction of Fluorescent-Labeled and Biosensor Bacterial Strains

The mNeonGreen (mNG)- and dsRed-labeled *P. fluorescens* SBW25 strains harbored an environmentally stable plasmid derivative that constitutively expresses the dsRed or mNG fluorescent proteins, as described (Wilton et al., 2017). *B. subtilis* strains labeled with the mCherry fluorescent protein were constructed by transferring the genetic construct P_*pen*_-mCherry:*kan* from *B. subtilis* MMB1023 (Babic et al., 2011) into the NCIB3610 background by SPP1-mediated phage transduction. The resulting NCIB3610-mcherry strain, was then used as a recipient for the transfer of sensory genetic elements. A xylose-responsive genetic module was constructed by inserting a P_*xylA*_ BioBrick DNA block (PCR-amplified from plasmid pBS1C3-PxylA) into plasmid pBS1C (Radeck et al., 2013) to form pBS1C-PxylA, which was then used as recipient for the insertion of the *gfp*_*sp*_ gene from pRD111 (Overkamp et al., 2013), using Spe1 and Pst1 restriction sites. In the final plasmid pBS1C-P_*xylA*_-gfp_*sp*_, the *gfp*_*sp*_ gene is under control of the xylose-inducible P_*xylA*_ promoter, linked with a chloramphenicol resistance gene (*cat*) and flanked by the right and left arms of the *B. subtilis amyE* gene. The P_*xylA*_-*gfp*_*sp*_:*cat* module was integrated in the *amyE* gene of *B. subtilis* strain 1A976 (SKC6) by transformation of the competent cells. A ROS-responsive *B. subtilis* strain was constructed by SPP1 transduction of the genetic module P*_*katA*_-gfp*:*spc* from strain 1A1010 (Bacillus Genetic Stock Center^1^), where P_*katA*_ is the promoter controlling the expression the catalase gene, in the NCIB3610 strain for integration at *amyE locus*. Transductants were selected for resistance to spectinomycin. Finally, the P*_*xylA*_-gfp_*sp*_* and P_*kat*_-*gfp* biosensing modules were transferred in the NCIB3610-mCherry strain by SPP1-mediated phage transduction.

### Characterization of Bacterial Biosensor Strains

In order to assess the specific response to xylose of the *B. subtilis* strain containing the *Pxyl:gfp* sensing module, cells were cultivated in a 96- well plate at 30°C in transparent minimal complete media supplemented with glucose (0.5%), xylose (0.5%), or arabinose (0.5%). Cell density (OD600) and green fluorescence (ex485/em520) were monitored in a multimodal microplate reader (Hidex Sense, Lablogic, Tampa, FL, United States). Relative fluorescence units (RFUs) were determined as culture fluorescence normalized to the cell density. The induction of GFP expression upon addition of a sugar source was calculated as the RFU difference between treated and untreated cultures. Fluorescence intensity was further corrected for cells auto-fluorescence by subtracting the average auto-fluorescence of NCBI3610 cells (without a *gfp* gene). The xylose biosensor cultures were imaged with a fluorescence microscope to assess signal homogeneity within the cell population. Aliquots were taken 5 h after the addition of xylose and stained with the red membrane stain FM4-64 (Invitrogen, Carlsbad, CA, United States) and immobilized on a layer of 1.3% of agarose-pad prior to imaging.

To assess the response to ROS of the NCBI3610 strain containing the *Pkat:gfp* construct, cells were cultivated in a 96- well plate at 30°C in transparent minimal complete media supplemented with 0.5% glucose as carbon source. Cultures were treated or not with H_2_O_2_ 0.015% (final concentration) and green fluorescence (ex485/em520) and cell density (OD600) were monitored over time. RFU were calculated and corrected for autofluorescence as described above. The distribution of fluorescence in the cell population was assessed by fluorescence microscopy.

### Cultivation of Aspen and Rice Seedlings in the RMI-Chip

*Populus tremuloides* Michx. seeds were cold stratified in Milli-Q water at 4°C for 2–14 days. Seeds were then surface sterilized by prewashing briefly in 1% Tween-20 and incubated in pH-reduced (10 mM HCl) 0.1M sodium hypochlorite. After 4 min of incubation the seeds were rinsed 8–10 times with sterile Milli-Q water and incubated in sterile Milli-Q water overnight in the dark at room temperature. The following day the seeds were spread onto 1% agar Johnson’s plate (4 mM KNO_3_, 2 mM Ca(NO_3_)_2_, 4 mM NH_4_NO_3_, 0.5 mM KH_2_PO_4_, 1 mM MgSO_4_, 440 μM KCl, 250 μM H_3_BO_3_, 20 μM MnSO_4_, 20 μM ZnSO_4_, 5 μM CuSO_4_, 5 μM NaMoO_4_, 5 μM CoCl_2_, 200 μM Fe, Na-EDTA, pH 5.6, 1% Phytoblend). The seeds were incubated under low to medium light on a 16 h light/8 h dark period, maintaining the temperature below 30°C. Once germinated, seedlings with hypocotyls shorter than 5 mm were transferred into cut 200 μL pipette tips filled with 1% Johnson’s agar to let the root elongate gravitropically. For a typical experiment, 20–25 pipette tips were inserted into Johnson’s agar (1%) plates at a ∼45° angle and incubated under the same conditions (Supplementary Figure S1). Root growth was checked periodically. Six seedling-containing tips with roots grown close to the end of the tips were mounted into the RMI-chip, which was incubated at a ∼45° angle to facilitate root infiltration. The measured photosynthetic photon flux density was ∼150 μmol/m^2^/s with a 16 h light/8 h dark period.

De-hulled rice seeds (*Oryza sativa* “Diamante-INIA”) were prewashed briefly in 1% Tween-20, followed by incubation in pH-reduced (40 mM HCl) 0.4M sodium hypochlorite solution on a rocker for 15–20 min. The seeds were rinsed seven times with Milli-Q water, plated on 1% Johnson’s agar, and incubated at 30°C in the dark for 48–72 h. When germinated and the radical protrusions reached 3–6 mm, the seedlings were planted directly into cut 200 μL pipette tips filled with Johnson’s solution already inserted into the RMI-chip. The measured photosynthetic photon flux density for rice was ∼250–300 μmol/m^2^/s with a 16 h light and 8 h dark period.

Following surface sterilization, all manipulations of seeds and seedlings were performed in a laminar flow hood, including mounting in the RMI-chip. Syringes, tubing, and adaptors were autoclaved prior to connection to the chip to maintain sterility during the extended continuous perfusion. The Johnson’s solution was filter-sterilized prior loading into autoclaved syringes.

### Bacterial Inoculation of the RMI-Chip

**FIGURE 1:**
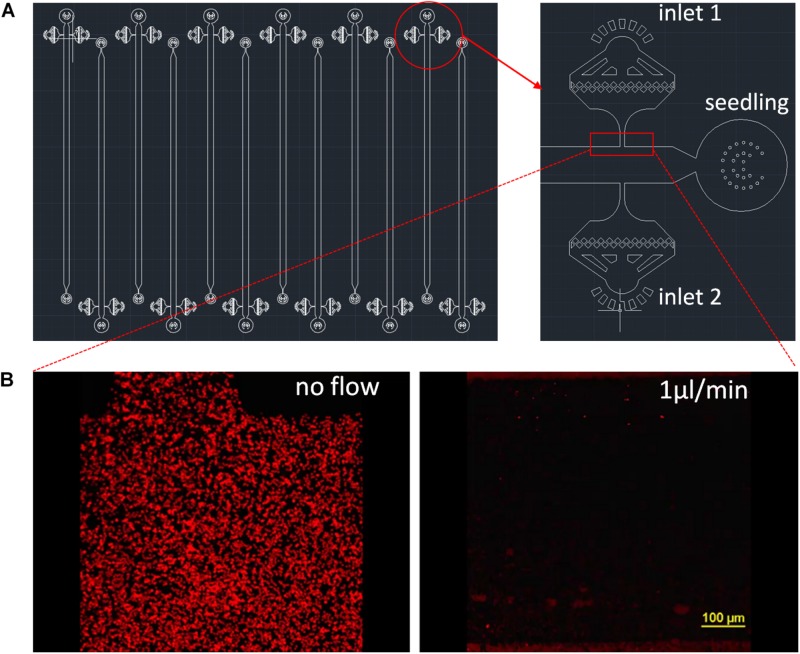
RMI-chip design. **(A)** The CAD design of the RMI-chip. A single growth channel is 36 mm long, 100 μm high, and 800 μm wide **(A)**. The media and inoculation inlets are equipped with on-chip filters to avoid occlusions during week-long experiments. Design files are available in the online supporting materials. **(B)** Flow dynamics. Fluorescently labeled latex beads were used to measure media flow in the empty RMI-chip. The root entrance and the inoculation inlets were plugged with stopper pins to mimic operational flow regime of the chip. The latex beads suspended in Johnson’s were injected and the chip was imaged without and with perfusion with Johnson’s solution at 1 μL/min. The latex beads were washed from the upstream chambers and the velocity of the beads were averaged. The measured and calculated values agreed. Laminar flow was observed under the microscope.

Bacterial species were cultivated overnight in LB at their respective optimal temperature (i.e., 28°C for *P. fluorescens*, 37°C for *B. subtilis*) in the presence of the appropriate antibiotic selection. Overnight cultures of *P. fluorescens* SBW25-mNG and -dsRed strains were centrifuged, rinsed in PBS, and re-suspended in PBS to OD_600_ = 4. A 10 μL aliquot of cell suspension was used for inoculation of aspen roots. Overnight cultures of *B. subtilis* biosensor strains were diluted 100-fold and grown in LB up to mid-exponential phase (OD_600_ = 0.4), rinsed with PBS and likewise adjusted to OD_600_ = 4 for the inoculation of the roots of aspen and rice seedlings. After overnight incubation without flow, excess bacteria were washed away with sterile Johnson’s solution at a flow of 0.2 μL/min for 2 h, followed by continuous perfusion at 0.02 μL/min. The outlet ports were connected to 200 μL pipette tips during inoculation, excess liquid was removed, and ports were connected to independent lines to prevent cross-communication between the 100 μM × 800 μm channels (Figure 1A) and with the external Johnson’s solution overlay during perfusion. Note that in theory, the RMI-chip can be washed and autoclaved after first use making the second set of six independent channels available for a new experiment. In this study, RMI-chips were used only once.

### Imaging Experiments

An inverted Nikon C2+ laser-scanning confocal microscope was used for imaging experiments (Nikon, Melville, NY, United States). An Eclipse Ti-E inverted microscope equipped with perfect focus system, an automated stage, and with 10×, 20×, and 100× objective lenses (CFI Plan Fluor 10×, NA 0.3, WD 16 mm; CFI Plan Apochromat Lambda 20×, NA 0.75, WD 1.00 mm; and CFI Plan Apo Lambda 100×, NA 1.45, WD 130 μm, respectively) was used for single image, time series, and z-stack acquisitions. Laser illumination emission at 488 nm coupled with a 525/50-nm excitation filter was used to capture mNeonGreen fluorescence, and laser illumination at 561 nm coupled with a 595/50-nm excitation filter was used to capture dsRed (*P. fluorescens*) or mCherry (*B. subtilis*) fluorescence. The transmitted light was also detected to image bacterial colonization in the context of the root structure. A custom holder was designed to provide access of the objective to the RMI-chip and imaging chamber.

## Results

### RMI-Chip Design Optimized for Aspen Seedling Growth

**FIGURE 2:**
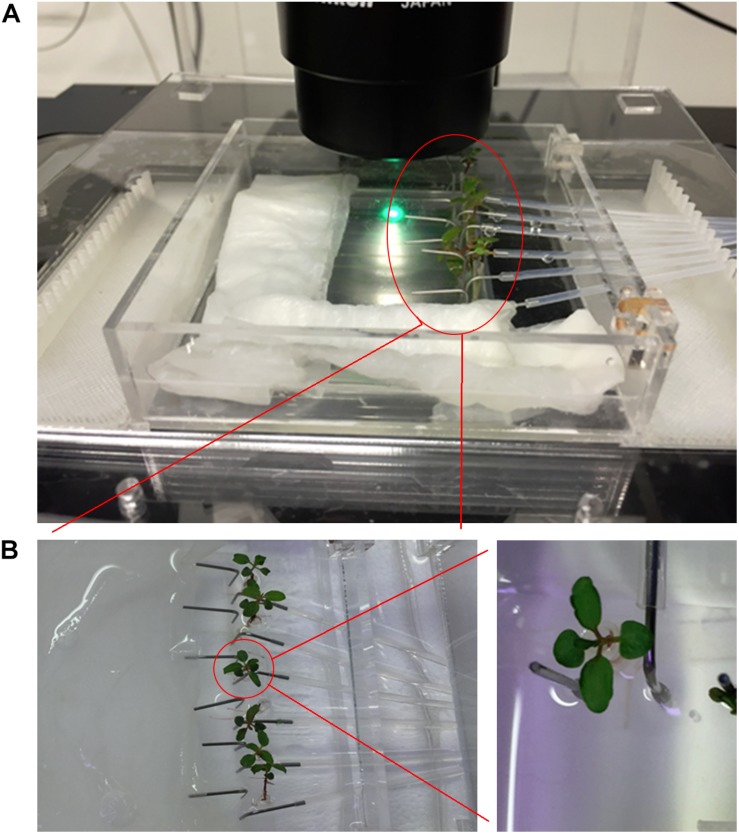
Imaging setup. **(A)** The imaging setup with humidity chamber and 3D printed stage adaptor. The RMI-chip has an inlet for media, a separate port for inoculation, and a wide channel to accommodate the tree seedling root without interfering with constant media flow. **(B)** The continuous flow incubation chamber enables the cultivation of six seedlings in parallel (left). Details of an aspen seedling (right).

The RMI-chip was designed to observe the growth and rhizobacterial colonization of aspen seedling roots over extended periods of time. The aspen root system consists of a taproot from which smaller branch roots emerge. When a seed germinates, the first root to emerge is the primary root which develops into the taproot. With seedlings grown into agar-filled pipette tips (Supplementary Figure S1a), we observed that the primary root quickly branched after exiting the tip (Supplementary Figures S1b, S2a). Thus, seedlings were inserted into the RMI-chip shortly before the primary root reached the end of the pipette tip. In early studies, we observed that although the primary root readily entered the RMI-chip circular chamber (Figure 1A), a substantial fraction of the roots did not continue into the linear growth channel, instead growing in circles and causing the seedling to wither quickly. This problem was resolved by growing the seedlings semi-gravitropically in a chip tilted at a 45° angle, submerged in Johnson’s solution for up to a week, until the root tip reached the media inlets in the growth channel (Supplementary Figure S2c). Under these conditions, most primary roots entered the growth channel, at which point the RMI-chip was placed horizontally in a humidity chamber and continuous media flow was supplied (Figure 2). Regular exchange of the nutrient solution surrounding the RMI-chip and the maintenance of water-saturated paper used to provide the required humidity in the growth chamber, were crucial to obtain healthy seedling growth.

The RMI-chip has six independent channels with one inlet for the seedling root, two dedicated inlets with on-chip 5 μm filters for media and bacterial inoculation, and one common outlet (Figure 1A). The features are generated via soft lithography on a silicon wafer, which is then used as a mold to produce PDMS slabs. The PDMS is bonded to a large thin microscope coverslip providing a means for imaging at cellular resolution using a confocal microscope. Different channel depths were tested for aspen seedling growth. Although aspen primary root can grow into an 80 μm × 80 μm channel in a submerged microfluidic device (Supplementary Figure S2b), the root occupied the entire channel precluding flow experiments. Therefore, we selected a channel width of 800 μm and fabricated channels with depths of 100 μm and 400 μm. While aspen seedlings grew similarly in both devices, the 400 μm deep channel did not position the root close to the glass coverslip, making it suboptimal for microscopic observations using objectives with standard working distances (Supplementary Figure S13). Our final design has growth channels 100 μm deep, 800 μm wide and 36 mm long, which can easily accommodate aspen primary roots and enable root growth under constant perfusion (Supplementary Figure S2c). Flow characteristics of the 400 μm × 800 channel were measured with fluorescently labeled styrene beads (Figure 1B). Without perfusion, slight bulk material movement was detected. At 1 μL/min, laminar flow was observed with a measured 50 μm/s bead velocity (Supplementary Videos S1, S2).

It can be challenging for plant roots to grow under constant flow. For example, without flow in a growth channel, root border cells and mucilage can be observed at the root tip (Supplementary Figure S3). These cells and mucilage can be washed away at high flow rates (e.g., 2 μL/min). Nevertheless, nutrient flow is needed to maintain seedling growth in the RMI-chip. Therefore, we determined a minimal flow rate of nutrient solution that did not affect root growth, preserving root morphology, including root cap, root hairs and mucilage, while keeping out air bubbles (Supplementary Video S3). After several iterations, each including at least 4 seedlings, the minimal flow rate was found to be 0.02 μL/min. As each channel is connected to an individual 1 ml syringe, this flow rate regime provides enough media for up to 5 weeks. Under these conditions, the calculated average laminar flow is 4 μm/s and the media is replaced every ∼14 min in each channel. We found that aspen seedlings exhibited a high heterogeneity in primary root growth with an average growth rate of 1 ± 0.9 mm/day (*n* = 10), resulting in some of the observed roots reaching the chamber exhaust after 10 days (Supplementary Figure S10) and some roots never reaching it during the 5 weeks of media flow. When a root blocks the media flow in the chamber, the seedling rapidly withers. In a fast-growing root, we also observed heterogeneity in the rate of root growth over time (Supplementary Figure S10). This heterogeneity may be due to the physical constraints imposed on the seedling by the RMI-chip confined space. For example, the chamber prevents the formation of lateral roots (Supplementary Figures S1, S2). Such a constraint on the root architecture could affect root growth in a manner that is not yet characterized. Another factor contributing to heterogeneity might be related to our use of open pollinated seeds which have different genetic makeups. Generally, only 4 or 5 seedlings out of 6 mounted in the RMI-chip typically sustained growth over a 5-week period. On average, aspen primary roots grew much slower than Arabidopsis primary roots reported to grow at 3.7 mm/day in the RootChip (Grossmann et al., 2011).

### Continuous Imaging of the RMI-Chip

Confocal laser scanning microscopy was used to perform spatial and temporal studies of the aspen root growth. Because of the repeated microscopy observations, a flexible system was required which was compatible with the transfer of the chip between the growth chamber and microscope stage. A modular chamber was designed to overcome this technical challenge and to prevent water evaporation during cultivation and imaging. An innermost imaging chamber was built to provide humidity during the repeated hour-long imaging experiments, and a medium-size chamber allowed submersion of the RMI-chip in media and its transport between incubation chamber and microscope. A six-channel syringe pump was connected with Teflon FEP tubing to the chip and transferred with the chip during imaging experiments in order to provide continuous flow of nutrients, even during long imaging experiments (Supplementary Figure S4). Finally, a large closed chamber that can be filled with water was built to provide humidity during long-term growth. In addition, a holder compatible with the imaging chamber was fabricated for the Nikon Ti-2 microscope (Supplementary Figure S4). The humidity chambers and microscope stage adapters were fabricated from PMMA thermoplastic. 3D printed inserts were fabricated to manage the tubing during imaging experiments (design files are available in Supplementary Material). With this system, the RMI-chip was continuously perfused with Johnson’s solution flowing at 0.02 μL/min, including during the lengthy observations under the microscope, thus minimizing the disturbance of seedling growth. Despite these precautions, maintaining the RMI-chip on the microscope stage during an imaging session may substantially change the illumination and temperature conditions, likely affecting seedling growth.

### Growing Rice Seedlings in the RMI-Chip

We tested the compatibility of the RMI-chip with other plant species such as rice. Rice seedling roots could be introduced easily into the chamber without an agar tip by directly transplanting the seedlings within days after germination. Healthy root growth was observed under continuous perfusion with Johnson’s solution at a 0.02 μL/min flow rate (Supplementary Figure S5). Under these conditions, we observed fast root growth where the root tip reached the end of growth chamber after 4 days on average. As for aspen, variability between seedlings was observed, some root tips reaching RMI-chip end after 3.5–5 days. Thus, rice root growth rate is approximately 10 times faster than aspen root growth under the same conditions. Unfortunately, the fast growth rate of rice seedlings limits observation time to 3–5 days. Once the root tip reaches the outlet, it blocks the chamber and cuts off nutrient flow.

### Colonization of Aspen Seedling Root by *P. fluorescens*

Aspen seedlings grown for 8 days in the RMI-chip were inoculated with mNeonGreen-labeled *P. fluorescens* SBW25 by injection of approximately 2 × 10^9^ cells through an inlet port (Supplementary Figure S6a). After incubation for 16 h without flow, the flow was restored at 0.2 μL/min for 2 h to wash away excess bacterial cells, then set at 0.02 μL/min for continuous perfusion. Under these conditions, a small number of SBW25 cells remained associated with the lower part of the root (Supplementary Figure S6b). Importantly, the only source of carbon available for bacterial growth in the RMI-chip are the sugars and organic acids photosynthetically produced by the plant. Five days after inoculation, actively dividing SBW25 cells were colonizing the intercellular spaces between root epidermal cells of the root cortex, and, to a lesser extent, the root surface (Supplementary Figure S7). Although this preferential colonization of crevices on the root surface may be caused by the flow in the RMI-chip, it is consistent with our previous observations in a vertical plate system, where SBW25 first colonizes the cell interstitial spaces along the aspen root cortex before forming a variety of bacterial assemblies that range from microcolonies to highly structured biofilms (Noirot-Gros et al., 2018).

**FIGURE 3:**
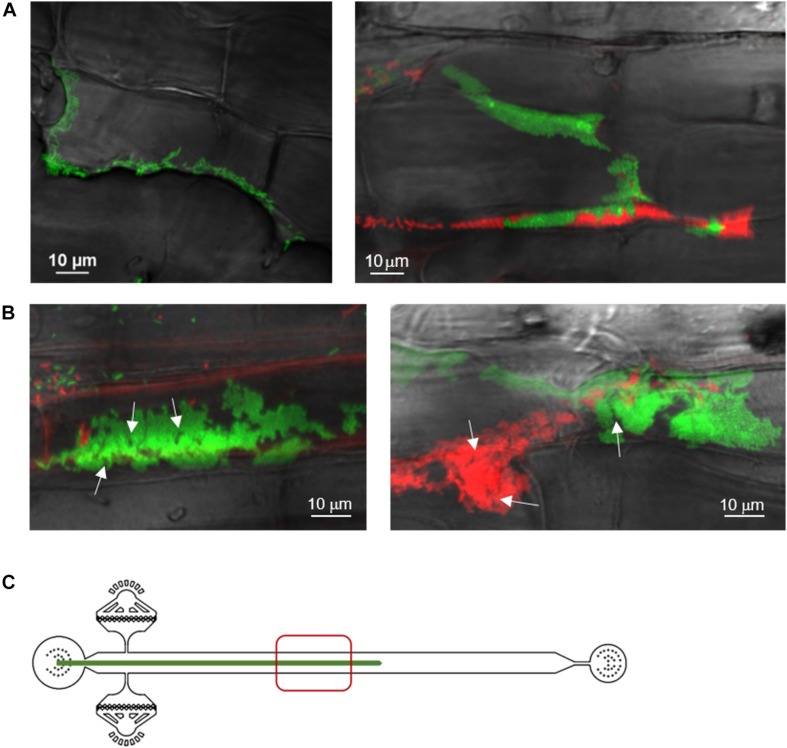
Spatial and temporal colonization of aspen primary roots by *P. fluorescens* in the RMI-chip. *P. fluorescens* SBW25 strains expressing dsRed or mNeonGreen were co-inoculated on aspen roots growing in the RMI-chip. **(A)** After 3 days, bacterial cells colonize aspen root surface by filling intercellular spaces (left panel) and adhering to plant cell surfaces (right panel). **(B)** After 13 days, SBW25 cells formed spatially segregated red and green long patches of cells that correspond to dense biofilm-like assemblies. Typical of mature biofilms, some internal void spaces and channels are indicated by arrows. **(C)** Schematic representation of a root in the RMI-chip with the zone (red square) scanned by the microscope to observe the bacterial assemblies.

Next, we observed aspen primary root colonization by a 1:1 mixture of two *P. fluorescens* SBW25 derivatives labeled with distinct fluorescent proteins, mNeonGreen and dsRed. Three days after inoculation, we observed discrete patches of red and green cells with almost no area of mixed colors (Figure 3A). This finding indicates that bacterial cells divide and form expanding patches in the intercellular spaces and on the root surface, ruling out that those patches are formed by random deposition and aggregation of cells within the root crevices. Thirteen days after inoculation, bacterial patches were longer and formed dense cell assemblies on the primary root surface (Figure 3B). Close-up examination revealed mature biofilms resulting from clonal growth of green and red bacterial populations. These biofilms exhibited channel-like void spaces, which are a hallmark of mature biofilms (O’Toole et al., 2000). Although cell density and presence of small aggregates in the pure cultures prior to co-inoculation could play a role in determining the colonization patterns (Melaugh et al., 2016), the quasi-absence of intermixed biofilms is indicative of a competition between the green and red strains. These strains have an identical genome but differ by the expressed fluorescent protein, possibly creating differences in cell fitness and some competition.

**FIGURE 4:**
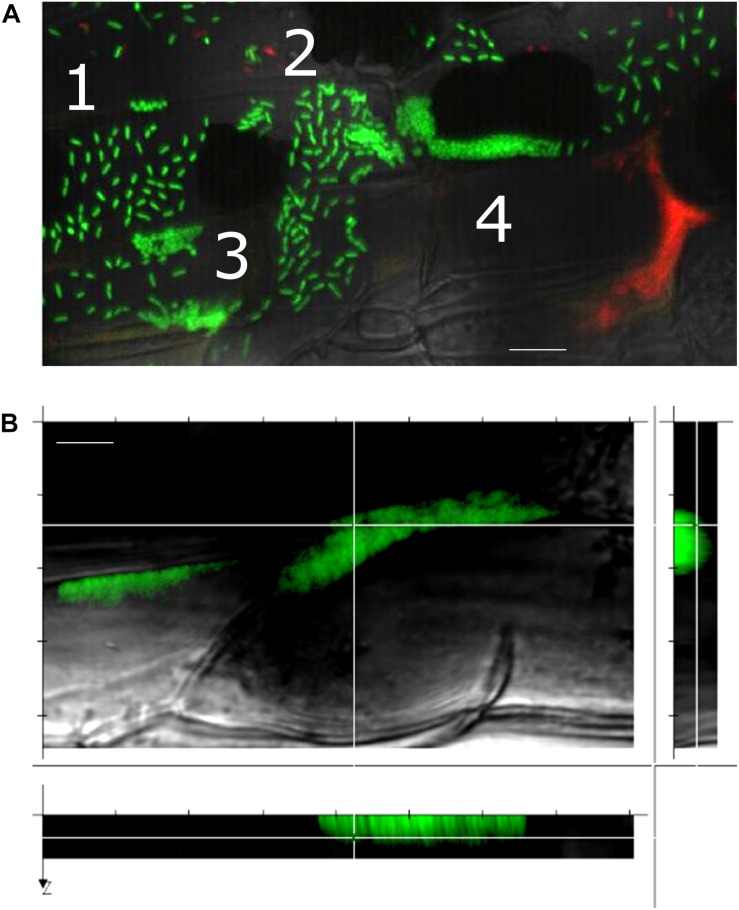
A gallery of bacterial assemblies on the root surface. **(A)** Image showing diverse assemblies of green SBW25 cells on the root surface: 1, individual cells; 2, cell doublets; 3, microcolonies; and 4, biofilms. **(B)** Projection of a reconstructed 3D volume with side views reveals a vertical alignment of cells within the biofilm. Scale bar indicates 10 μm length. Observations were performed in a root zone similar as that shown in Figure 3.

Notably, we observed that a variety of SBW25 cell assemblies coexisted with biofilms on the root surface (Figure 4A). These assemblies included clusters of somewhat regularly spaced individual cells, clusters of cell doublets which are presumably dividing, microcolonies and mature biofilms (Supplementary Figure S8). These assemblies may reflect the different stages of biofilm formation, where cells attach to the root surface, divide, and over time form more compact assemblies maturing into biofilms. Intriguingly, cells packed within a biofilm appeared to have a round coccoid shape in contrast with the normal rod-like morphology of individual SBW25 cells (Figures 3A, 4A). Confocal 3D image reconstruction and side projection of the 3D volume (Figure 4B) revealed that the coccoid shape was only apparent and resulted from the top view of vertically arranged bacterial cells within the biofilm. This finding corroborates our previous observation of vertical arrangements of SBW25 cells within biofilms and mucilage formed on the roots of aspen seedlings growing in a vertical agar plate (Noirot-Gros et al., 2018).

After 30 days in the RMI-chip, fluorescent SBW25 cells became rare on the root surface and were preferentially found as micro-colonies located within the spaces between root epidermal cells (Supplementary Figure S9). Overall, the various observable phenotypes in SBW25 assemblies, which include active cell growth, formation of cell patches on the root surface during the first 2 weeks, formation of mature biofilms associated with a variety of other cell assemblies, and dispersal of these biofilms and assemblies after 5 weeks, appear to be qualitatively comparable in the RMI-chip and vertical plate assay, the latter being a very different set-up where seedling roots grow on a nutrient agar surface (Noirot-Gros et al., 2018). This similarity suggests that the observed dynamics of cell assemblies are not primarily shaped by the flow in the RMI-chip but rather reflect an intrinsic colonization behavior of aspen primary root by *P. fluorescens* SBW25. In addition, novel cell assemblies such as clusters of somewhat regularly spaced individual cells and cell doublets were observed uniquely in the RMI-chip.

### RMI-Chip and Rhizobacterial Biosensors to Monitor Dynamic Root Exudation

In the RMI-chip, the carbon sources available for bacterial growth are provided by root exudates. These exudates are compositionally complex and contain ions (i.e., H^+^), inorganic acids, oxygen, water, and multiple carbon−based compounds which include amino acids, organic acids, sugars, phenolics and an array of secondary metabolites, and high−molecular weight compounds like mucilage and proteins (Badri and Vivanco, 2009). Root exudates are key mediators of interactions with microbes in the rhizosphere and root exudation is a regulated process which responds to biotic and abiotic stresses to the plant (Badri and Vivanco, 2009). We assessed whether whole-cell biosensors, which are engineered rhizobacteria that can express a fluorescent protein in response to the presence of a specific metabolite or environmental stressor, could be used in the RMI-chip to monitor dynamic changes in the composition of plant exudates.

We used *B. subtilis*, which colonizes Arabidopsis roots (Massalha et al., 2017) and is used as biocontrol agent to colonize and protect various herbaceous plants (Allard-Massicotte et al., 2016), to develop biosensor strains. We selected two well characterized promoters, one responding to the presence of xylose (Kim et al., 1996), a sugar abundant in root exudates, and the other one responding to the presence of ROS (Hoover et al., 2010), which are normal products of plant cellular metabolism and act as second messengers in plant responses to various environmental stresses (Sharma et al., 2012). Characterization of these biosensors in the laboratory (see section “Materials and Methods”) revealed that the xylose biosensor responded to the presence of xylose but not of glucose or arabinose (Supplementary Figure S12a) and displayed homogeneous expression in the cell population (Supplementary Figure S12b). The ROS biosensor was induced by the presence of 0.015% H_2_O_2_ (Supplementary Figure S12c) but displayed a heterogeneous expression in the cell population (Supplementary Figure S12d). Note that the biosensors deposited on the roots also expressed constitutively the red fluorescent protein mCherry, which serves to label the cells (see Experimental methods).

**FIGURE 5:**
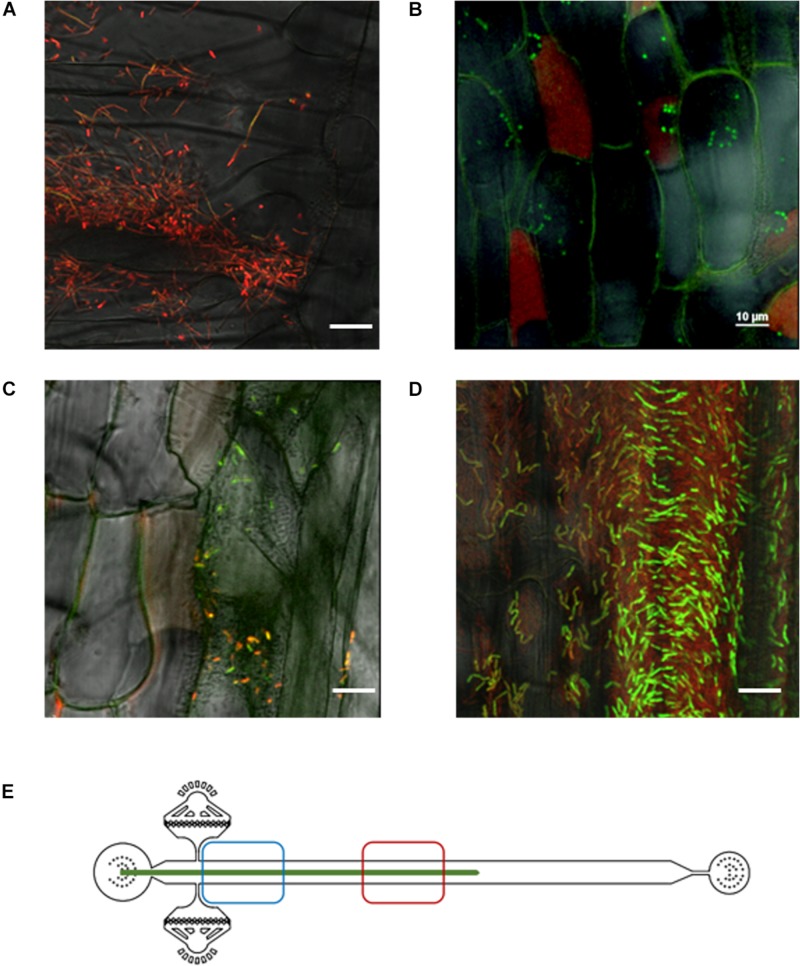
*Bacillus subtilis* biosensors exposed to aspen root **(A–C)** and rice root **(D)** in the RMI-chip. **(A)** Among the root hairs, the xylose biosensor cells appear red from constitutive mCherry expression with a very faint GFP (green) signal detected in some cells. Similar results were obtained with the ROS biosensor (not shown). **(B)** Five days after inoculation, rare attached biosensor cells overwhelmingly express GFP, indicating the presence of xylose in root exudates. Note the plant tissue autofluorescence causing cell outlines to be green and some plant cells to be entirely red. **(C)** After 5 days, most of the rare ROS biosensor cells expressed GFP, indicating the presence of ROS produced by the root. Plant tissue autofluorescence is also detected. **(D)** Three days after inoculation of a rice seedling, the *B. subtilis* ROS biosensor displayed robust colonization of the root with a strong expression of GFP in a substantial fraction of the cell population, indicating production of ROS by the rice root. Scale bar indicates 10 μm length. **(E)** Xylose- and ROS-sensing *Bacillus* cells in panels **(B)** and **(C)** were observed in the lower part of the aspen root (red square). ROS-sensing *Bacillus* cells on rice roots in panel **(D)** were observed in the upper part of the RMI-chip (blue square).

Aspen primary roots were inoculated with *B. subtilis* biosensors in the RMI-chip, incubated overnight without flow, and upon restoration of flow, the root was observed using confocal laser microscopy. We found that, as with *P. fluorescens*, most cells were rapidly washed away from the root surface. However, biosensor cells were very sparse on the root after 3 days and could not be observed after 5 days. The use of a low flow (0.02 μL/min) throughout inoculation of the RMI-chip did not enhance the number of biosensor cells remaining attached to the primary root, reflecting an inability of *B. subtilis* to colonize aspen primary root under these conditions. This is in contrast with our observations with *P. fluorescens* and may reflect the fact that *B. subtilis* is not a natural colonizer of aspen roots. To circumvent this problem, observations were done in a stopped-flow experiment limited to 5 days, after which the aspen seedling starts to wither. Even in the absence of flow, *Bacillus* cells did not colonize but remained loosely attached to the aspen root surface. Whereas biosensor cells did not express GFP after inoculation (Figure 5A), strong GFP signals were detected in biosensor cells remaining attached to the aspen root after 5 days of incubation, indicating the production of xylose (Figure 5B) or ROS (Figure 5C) by the distal part of the root (Figure 5E). Exposure of the ROS biosensor to a rice primary root under the same RMI-chip conditions (i.e., no flow) resulted in a robust colonization of the proximal part of the root and in high levels of GFP in a fraction of the cell population after 3 days (Figures 5D,E). This finding indicates that despite the intrinsic heterogeneous expression of our biosensor (Supplementary Figure S12d), high levels of ROS are produced by rice primary root under these conditions. Together, these results provide a proof-of-concept that bacterial biosensors can be used in the RMI-chip as a way to investigate the dynamic chemical crosstalk between root and rhizobacteria.

We also inoculated rice seedlings with *P. fluorescens* SBW25 to test for ability to colonize in the RMI-chip. Repeated trials showed that SBW25 cells were rapidly washed away from the root surface and remained barely detectable under slow nutrient flow after 1 day (Supplementary Figure S11), suggesting an inability of *P. fluorescens* SBW25 to colonize rice primary root under these conditions. This result was unexpected as rice was shown to host *Pseudomonas* endophytes (Jha and Subramanian, 2014). These observations clearly indicate that biosensors need to be developed from bacterial isolates that efficiently colonize the roots of the plant host of interest. What may seem to be a caveat for the development of biosensors is actually grounded in ecology and evolution, as root exudates from a particular plant species are known to maintain and support a highly specific diversity of microbes in the rhizosphere of this plant (Badri and Vivanco, 2009).

## Conclusion

In summary, we report the design and fabrication of a microfluidic device and accessories that enable the cultivation of aspen seedlings under constant nutrient flow and the study of RMIs through repeated functional imaging during a 5-week long experiment. The device enabled imaging at single bacterial cell resolution during the various phases of aspen colonization by *P. fluorescens* SBW25. Different biofilm morphologies and diverse bacterial assemblies were observed over time, emphasizing the need for high-resolution imaging to understand colonization patterns and strategies and their relation to the local root environment. *B. subtilis* whole-cell biosensors provided a means for functional imaging of bacterial cells actively consuming xylose from root exudates and actively responding to ROS, an environmental stressor produced by the root. However, the use of these biosensors to image compositional changes in the root environment was limited by the inability of *B. subtilis* biosensor to colonize and persist on aspen roots, suggesting that whole-cell biosensors should be built from naturally colonizing bacteria. The current design of the RMI-chip can be used for the long-term observation of slow-growing plants, or can be modified to study faster growing plants as well.

## Data Availability Statement

All datasets generated for this study are included in the article/Supplementary Material.

## Author Contributions

GB, M-FN-G, and PN designed the experiment and wrote the manuscript. GB and JJ designed and fabricated the RMI-Chip, humidity chamber, and imaging microscope stage. M-FN-G, RW, SZ, and KK developed fluorescent bacterial strains. SS, CA, JJ, and GB developed the protocols to adapt and grow healthy plants in RMI-chip and observed them over long periods. M-FN-G and GB analyzed RMI-chip images. All authors contributed to manuscript preparation, editing, and gave final approval for publication.

## Conflict of Interest

The authors declare that the research was conducted in the absence of any commercial or financial relationships that could be construed as a potential conflict of interest.

## Footnotes

1 http://www.bgsc.org
