# Supplementary material for: Functional Imaging of Microbial Interactions With Tree Roots Using a Microfluidics Setup
Source: Front Plant Sci. 2020 Apr 15;11:408. doi: 10.3389/fpls.2020.00408 (PMC7174594; doi:10.3389/fpls.2020.00408)
Supplement: Supplementary file 3 [file Presentation_1.pdf]

Supplementary Material

Functional imaging of microbial interactions with tree roots using a microfluidics setup

Marie-Francoise Noirot-Gros[1], Shalaka V. Shinde[1,2], Chase Akins[1], Jessica L. Johnson[1], Sarah Zerbs[1], Rosemarie Wilton[1], Kenneth M. Kemner[1], Philippe Noirot[1], Gyorgy Babnigg[1*]

[1]Argonne National Laboratory, Biosciences Division, Lemont, IL, USA

[2]Current address: Oil-Dri Innovation Center, Vernon Hills, IL, USA

*** Correspondence:** gbabnigg@anl.gov

Supplementary Figures and Table:

Figure S1. Aspen seedling cultivation in pipette tips.

Figure S2. Aspen seedling primary root entering the RMI-chip channel.

Figure S3. Tips of a growing aspen root in absence of flow.

Figure S4. RMI-Chip imaging and humidity chambers.

Figure S5. Rice seedling in the RMI-chip.

Figure S6. Inoculation of fluorescently labeled *P. fluorescens* in the RMI-chip

Figure S7. Colonization of aspen primary root by *P. fluorescens* SBW25.

Figure S8. Cell assemblies formed by *P. fluorescens* SBW25.

Figure S9. Long-term root-microbe interaction.

Figure S10. Example of an aspen root growing in the RMI-chip.

Figure S11. Lack of colonization of rice root with fluorescently labeled *P. fluorescens*.

Figure S12. Characterization of *Bacillus subtilis* sensor strains.

Figure S13. Imaging root growth in a 400 µm-deep RMI-chip chamber.

Video S1 and S2. Flow characteristics of the 400 μm x 800 channel section of the RMI-Chip.

Video S3. Flow of nutrient into the RMI-Chip at 0.02 μL/min.

Table S1. Oligonucleotides, plasmids and strains.

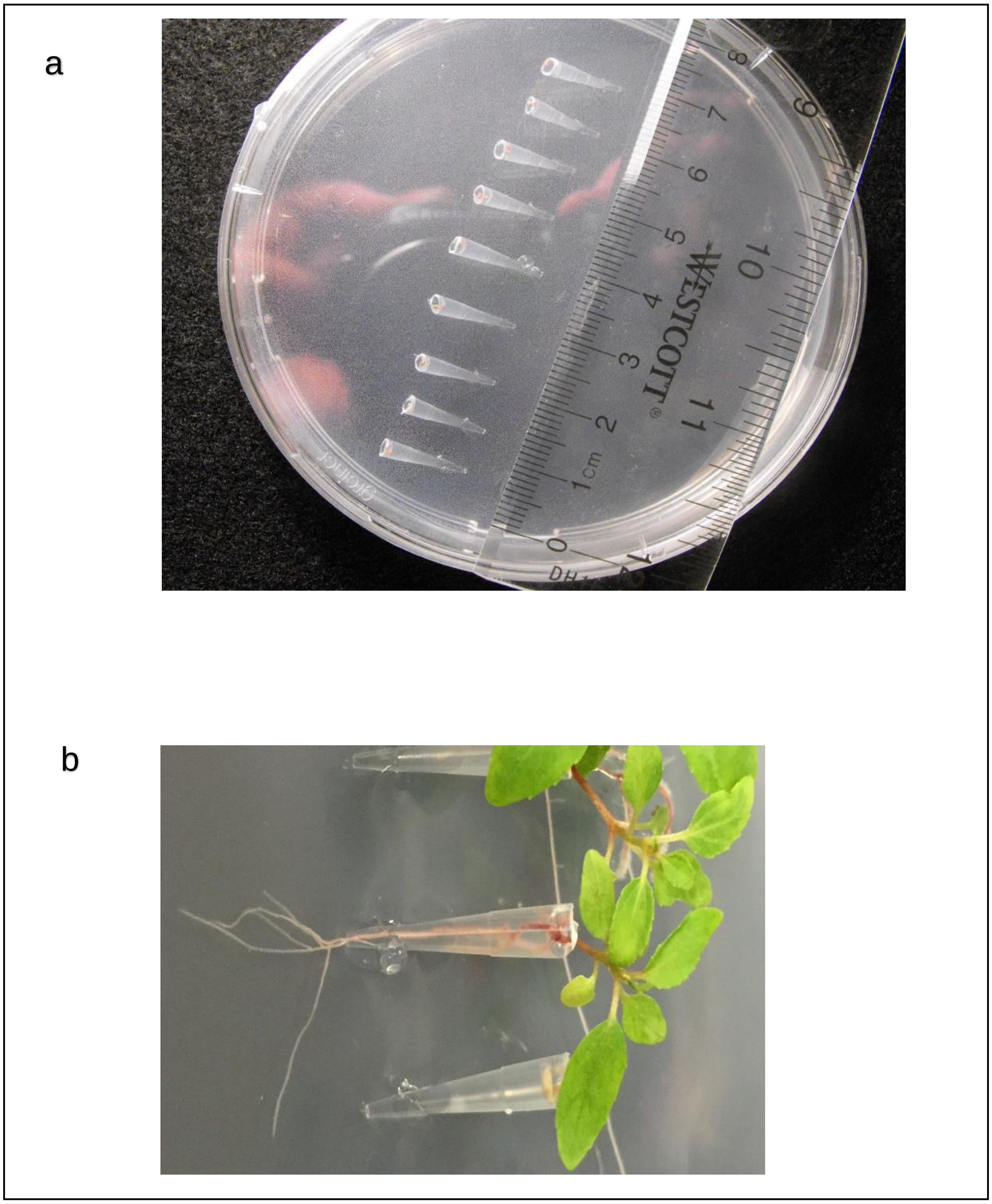

Supplementary Figure S1. Aspen seedling cultivation in pipette tips. (a) Aspen seeds were germinated and seedlings with < 5 mm hypocotyls were transferred into 200 µL pipette tips filled with 1% Johnson's agar. Tips were inserted at a 45° angle in a Johnson's agar plate for gravitropic root growth and incubated in a growth chamber. (b) Healthy plant growth was observed in the pipette tips and upon exit, the primary root quickly developed branch roots (center seedling). Tips selected for mounting into the RMI-chip had a primary root reaching the end of the pipette tip (top and bottom seedlings).

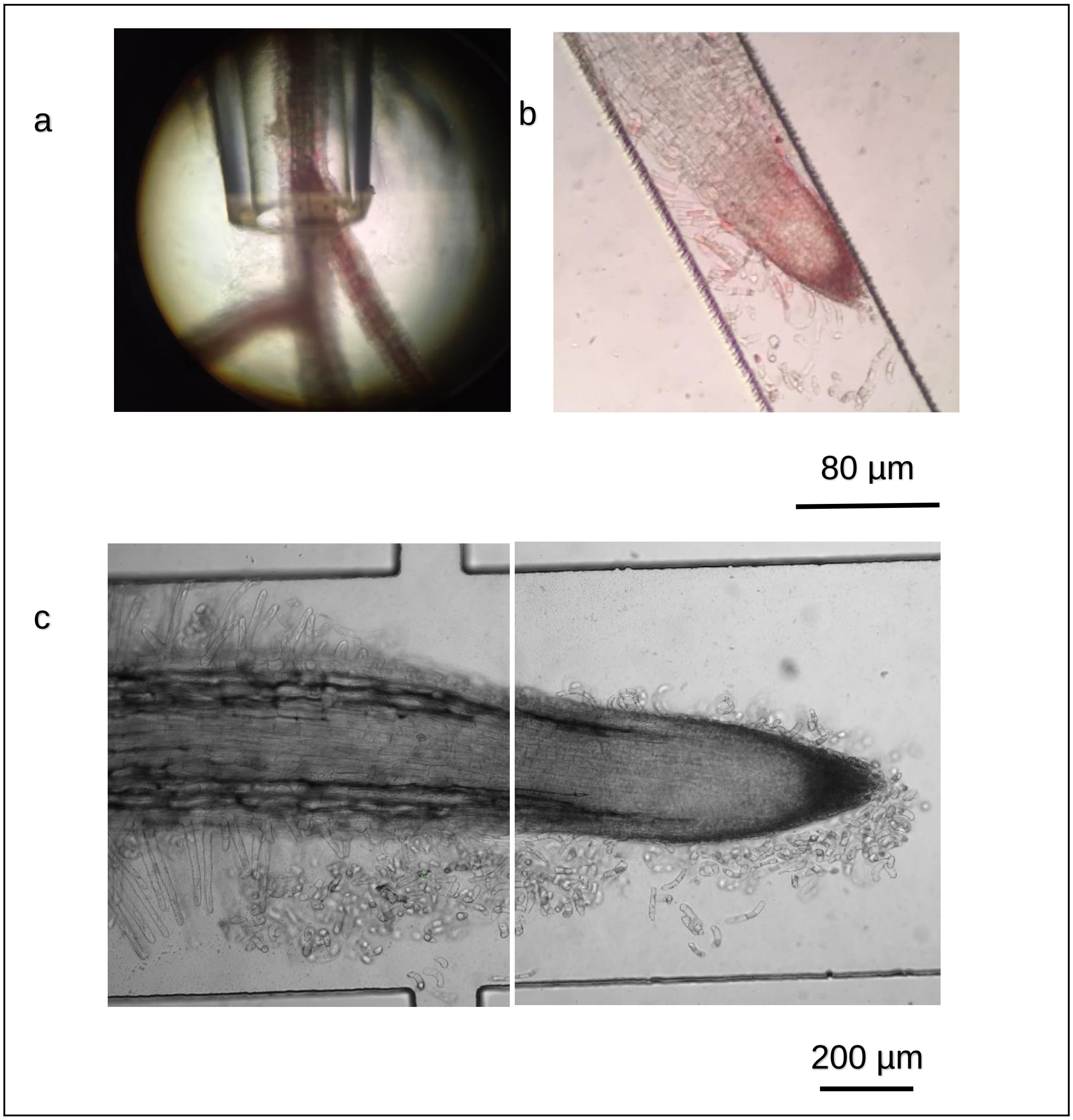

Supplementary Figure S2. Aspen seedling primary root entering the RMI-chip channel. (a) Normal root morphology was observed in the 200 µL pipette tips. Regular primary root development was followed by branch root formation when maintained in the Petri dish with Johnson's 1% agar. (b) Growth of the primary root was observed into a microfluidic channel of 80 µm x 80 µm where the root occupies ~~all~~ the channel space (right). Under these conditions, root growth stopped after a few days. (c) Primary root growing semi-gravitropically in a 100 µm x 800 µm channel of the RMI-chip tilted at a 45° angle and submerged in Johnson's solution. Within a week after mounting, the root tip reached the media inlets in the growth channel. Root hair, border cells and cell debris can be observed. At this stage, the RMI-Chip can be positioned horizontally and the flow of nutrient solution initiated.

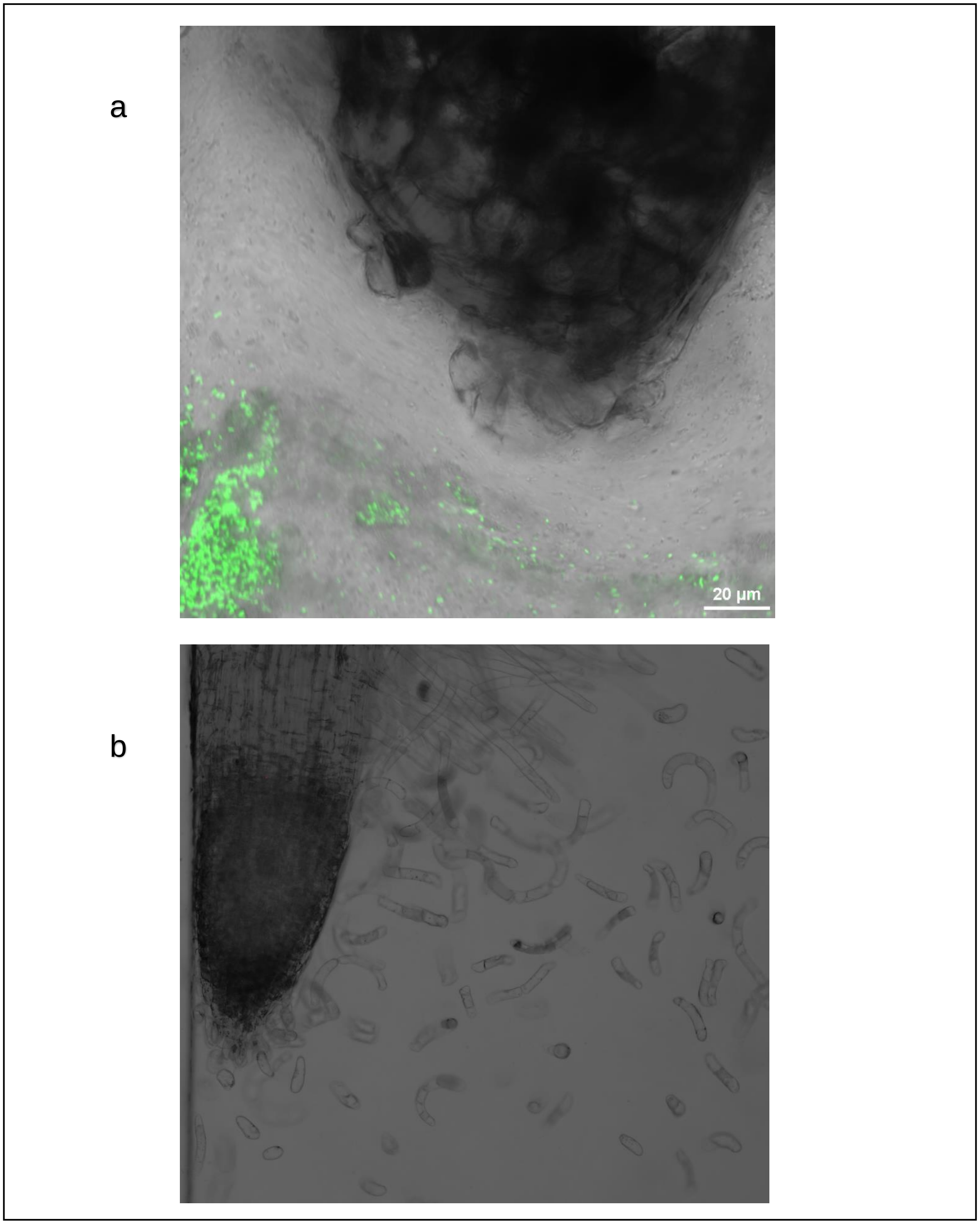

Supplementary Figure S3. Tips of a growing aspen root in absence of flow. (a) Mucilage formed in a closed channel prevents the direct interaction of aspen seedling root tip with mNeonGreen-labeled *P. fluorescens* SBW25. (b) Aspen seedling root border cells are observed in the RMI-Chip without perfusion.

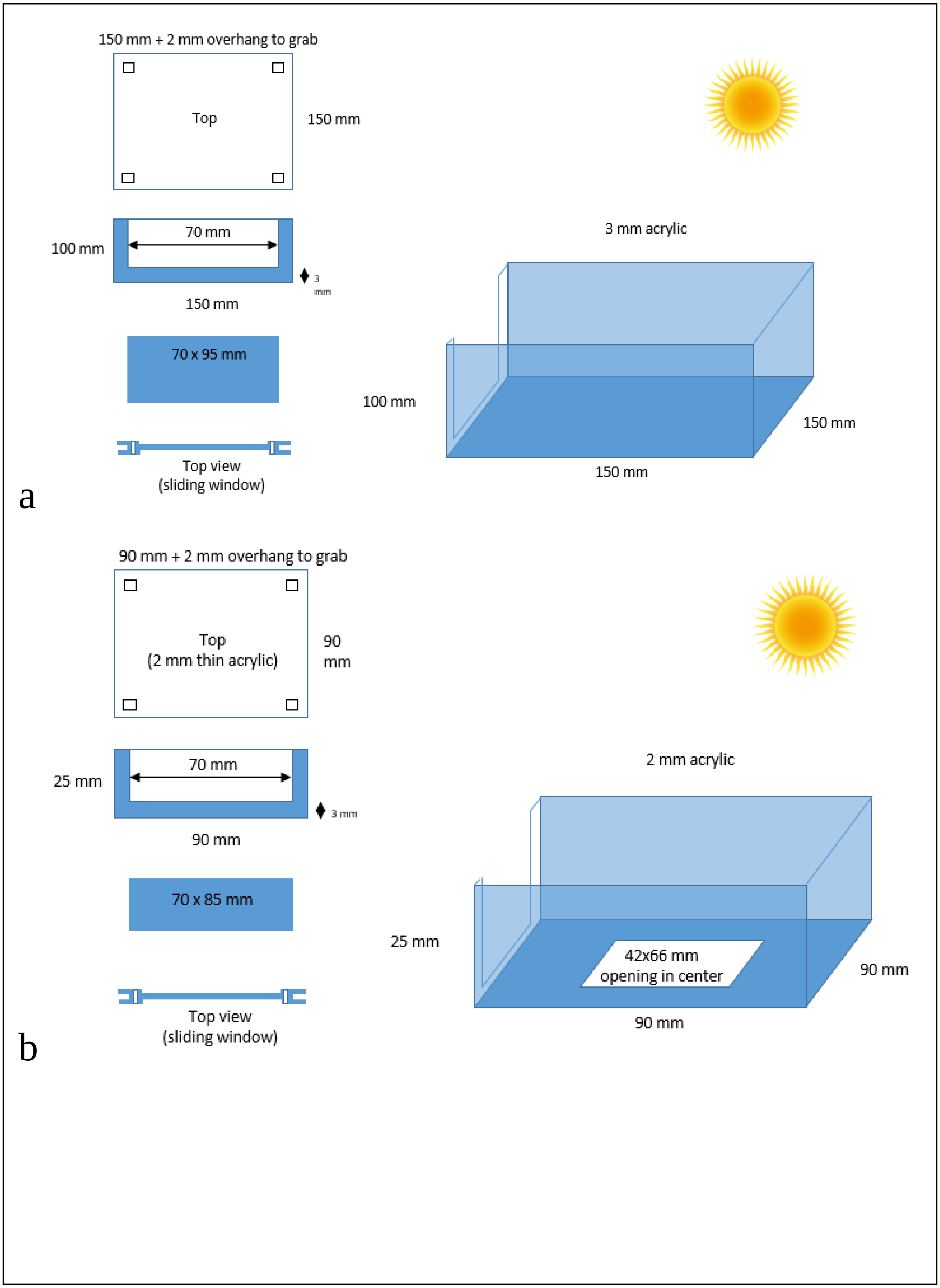
150 mm + 2 mm overhang to grab
Top
150 mm
100 mm
70 mm
150 mm
70 x 95 mm
Top view
(sliding window)
3 mm acrylic
100 mm
150 mm
150 mm
a
90 mm + 2 mm overhang to grab
Top
(2 mm thin acrylic)
90
mm
25 mm
70 mm
3 mm
90 mm
70 x 85 mm
Top view
(sliding window)
2 mm acrylic
25 mm
42x66 mm
opening in center
90 mm
90 mm
b

c

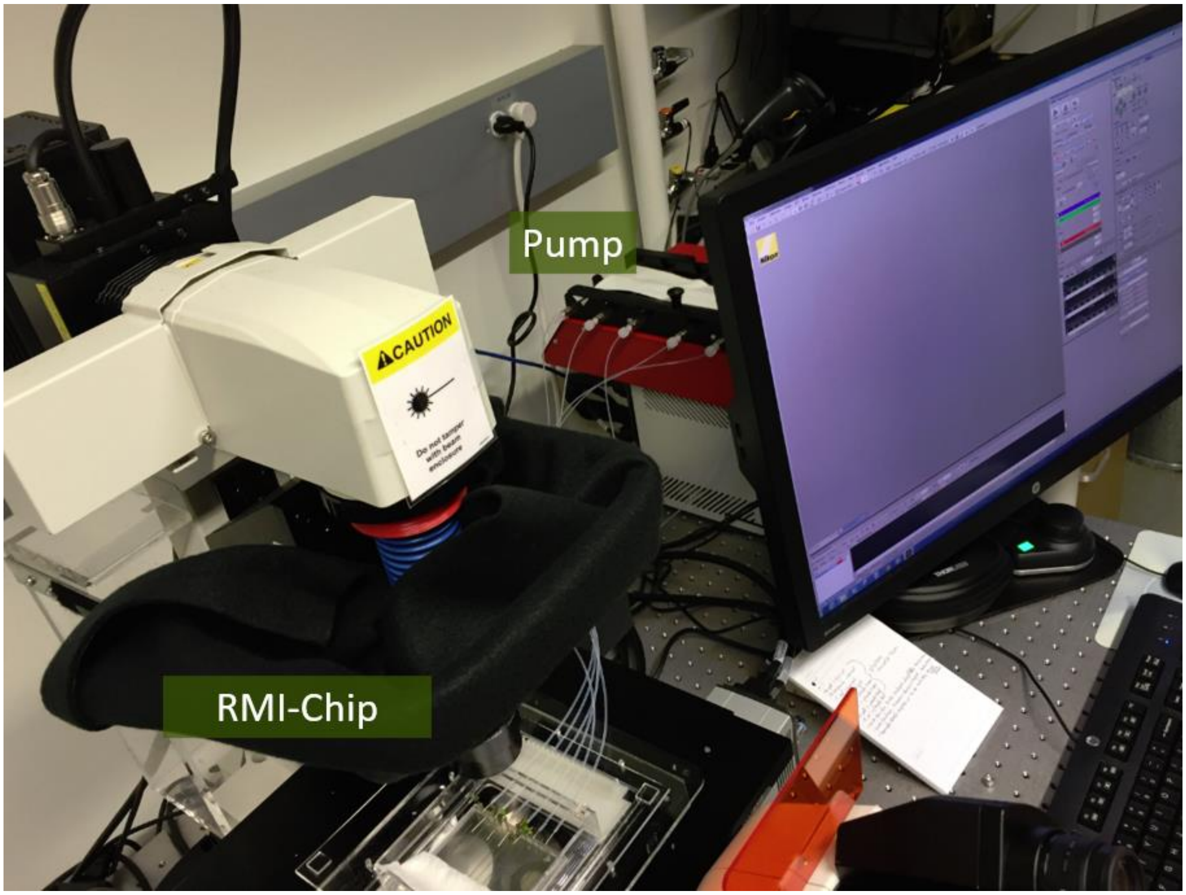

Supplementary Figure S4. RMI-Chip imaging and humidity chambers. (a) The humidity chamber is constructed from black PMMA side panels via laser cutting. A sliding side window enables the removal of the imaging chamber with tubing attached. (b) The imaging chamber has a bottom opening for direct high-resolution observations. (c) The modular setup with sliding windows enables continuous perfusion via a six-channel microfluidic pump connected to the RMI-Chip with FEP tubing. The imaging module is transferred with the chip during the imaging experiment.

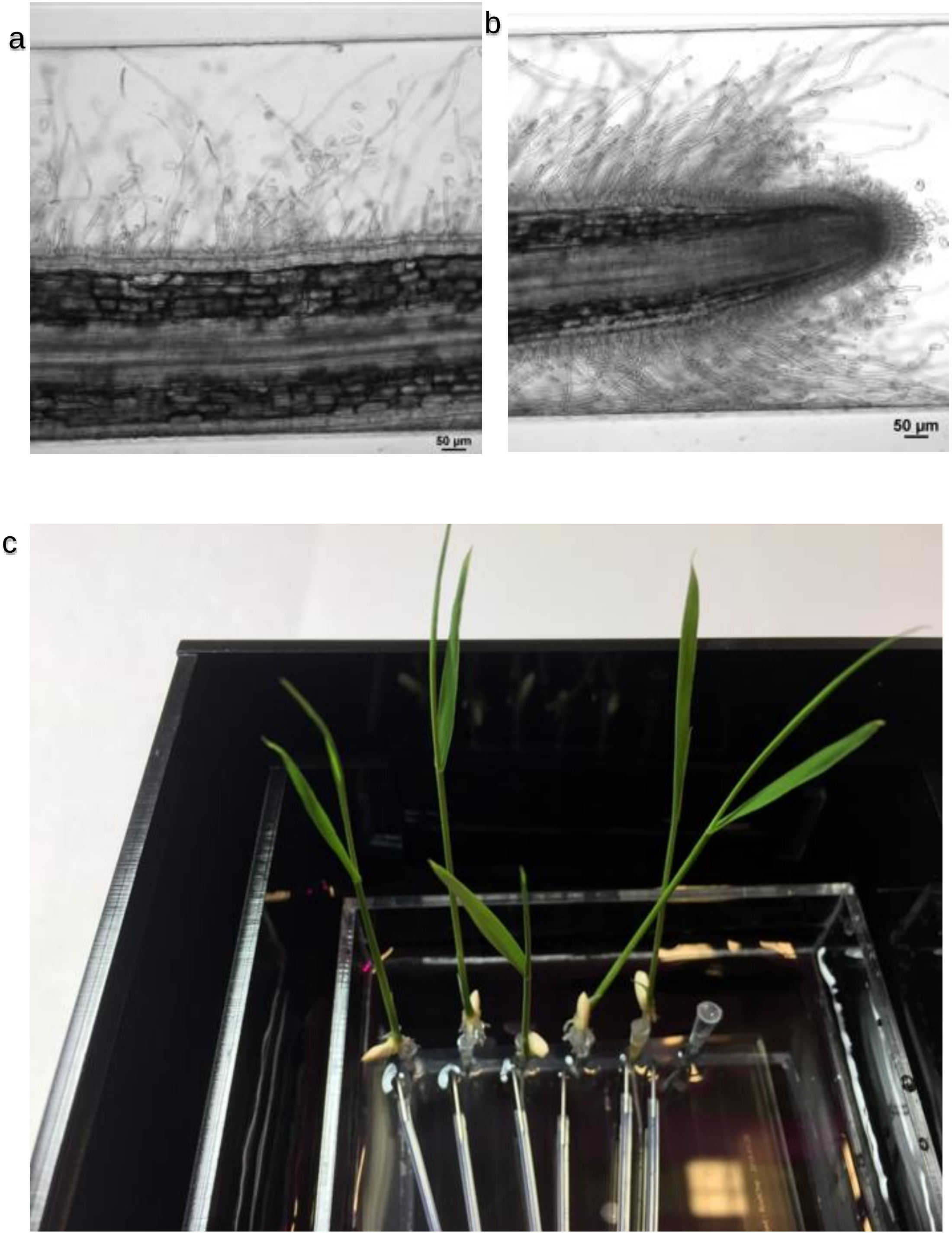

Supplementary Figure S5. Rice seedling in the RMI-chip. (a, b) Rice seedlings grew well in the RMI-chip enabling high-resolution observations of the root. (c) The 3-chamber design is ~~shown~~ for long-term cultivation and repeated imaging experiments under constant perfusion with Johnson's media.

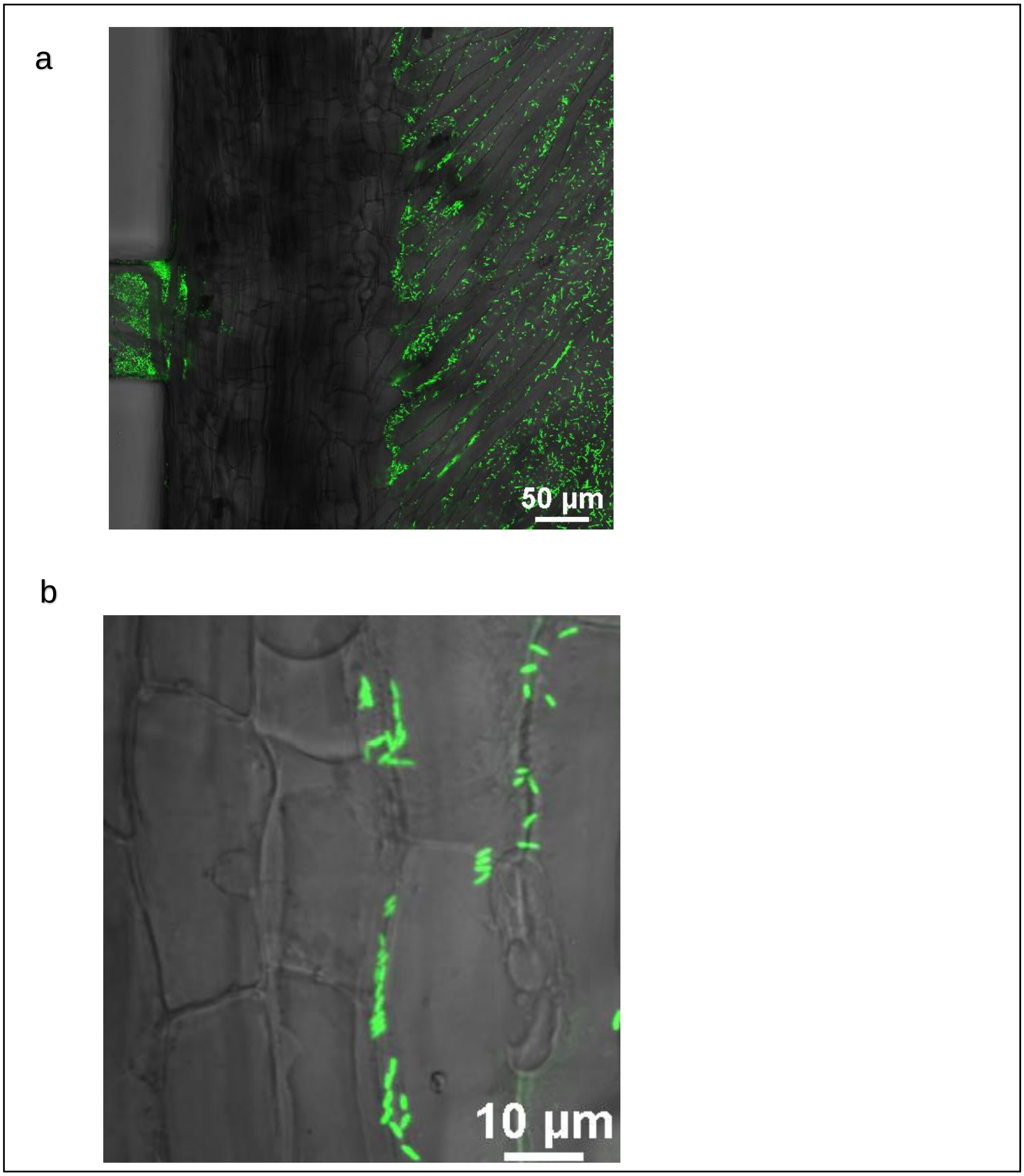

Supplementary Figure S6. Inoculation of fluorescently labeled *P. fluorescens* in the RMI-chip (a) SBW25 cells constitutively expressing mNeonGreen were injected through the inlet and incubated 16 hours without media flow. The bacterial population appears well dispersed with some concentration of cells at the base of aspen root hairs. (b) After continuous media flow was established (see Experimental procedures), most of the bacterial cells were removed. After one day of flow, only a small number of SBW25 cells remained associated with the lower part of the primary root.

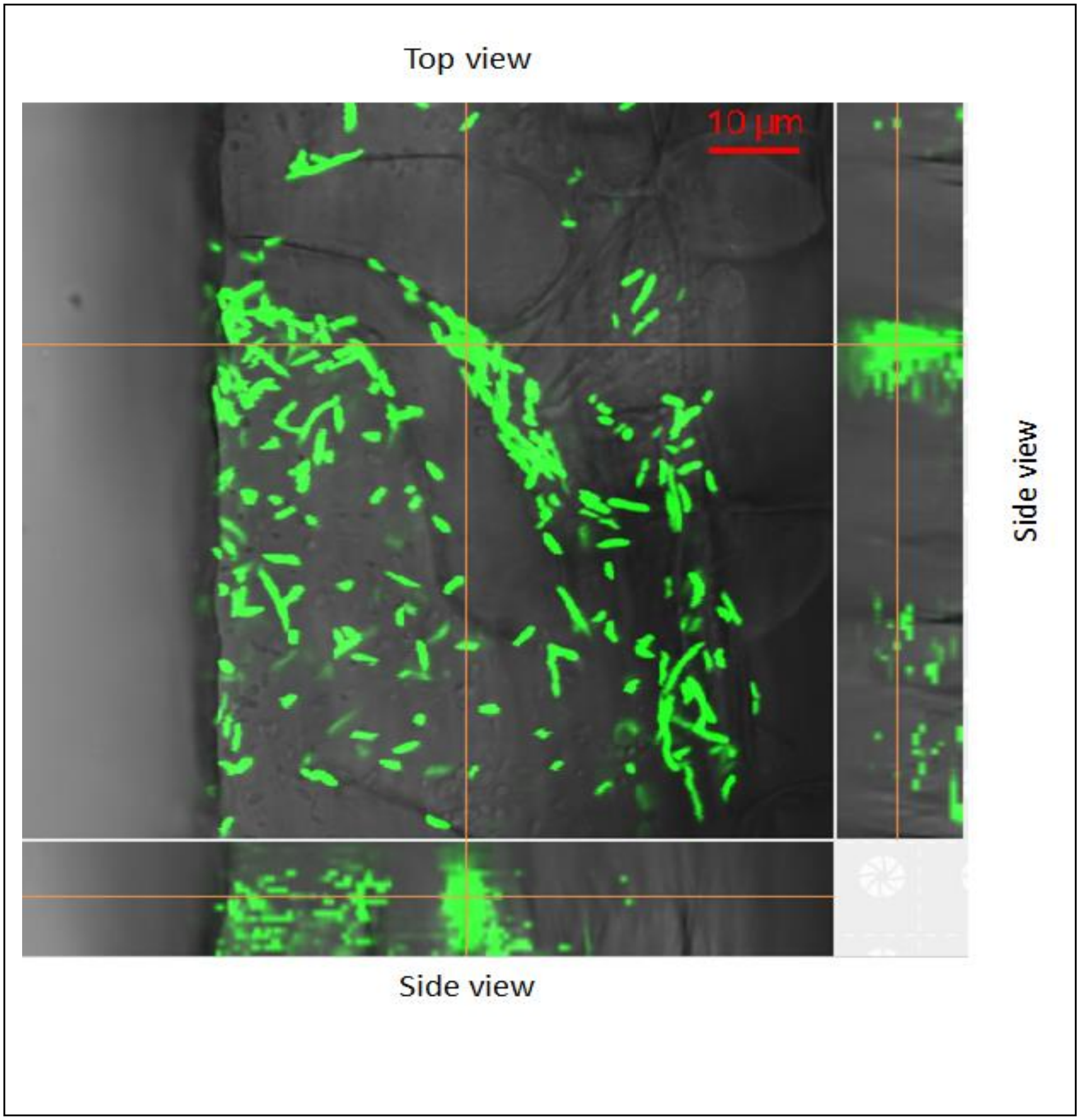

Supplementary Figure S7. Colonization of aspen primary root by *P. fluorescens* SBW25. Five days after inoculation, mNeonGreen-labelled SBW25 cells were actively dividing and colonizing the intercellular spaces between root epidermal cells of the root cortex, and to a lesser extent, the root surface. Projection of a reconstructed 3D volume with side views show preferential occupation of deep crevices on the root surface.

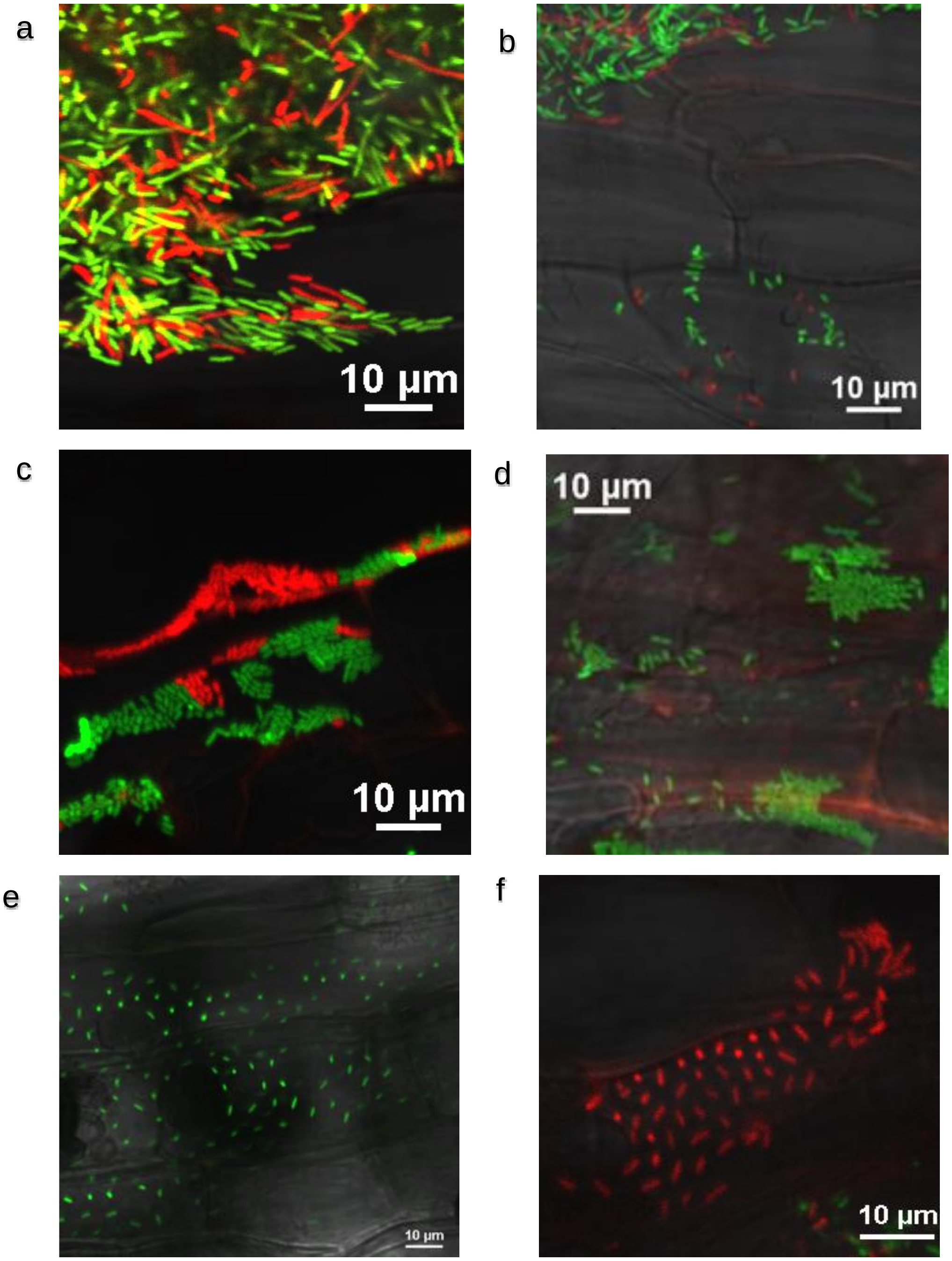

Supplementary Figure S8. Cell assemblies formed by *P. fluorescens* SBW25. (a) The inoculum including a mixture of mNeonGreen- and dsRed-labelled *P. fluorescens* SBW25 is mostly composed of elongated motile cells. (b) One day after inoculation and under flow, the root-attached cells become less elongated. (c) 13 days after inoculation, colonies of the labeled cells were observed, some with round shape microbes, resulting from tight packing. (d) Mixture of patches of microbes and loose assemblies are also observed in some regions. (e, f) Individual cells also formed quasi regularly spaced assemblies, irrespective of the label. The 3D confocal imaging revealed that the apparent coccoid shape in contrast with the normal rod-like shape of individual SBW25 cells resulted from the top view of vertically aligned cells.

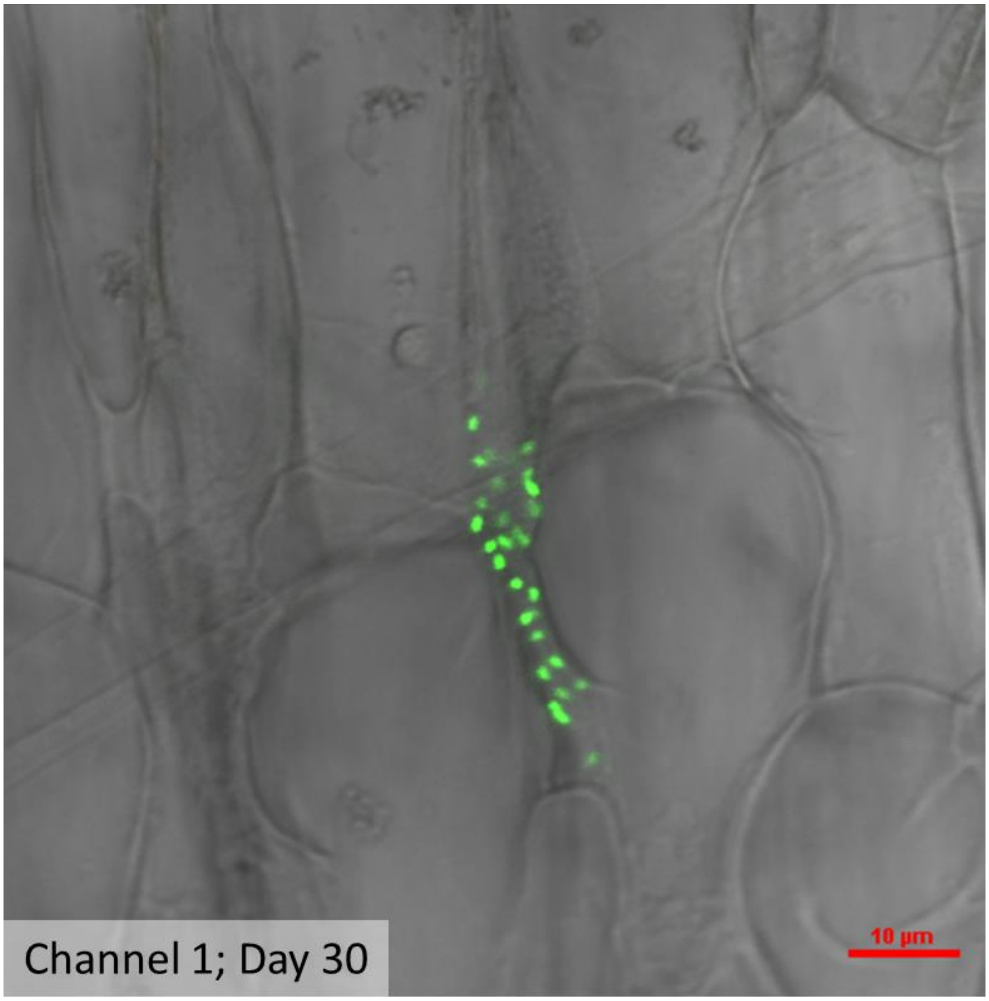

Supplementary Figure S9. Long-term root-microbe interaction. Fluorescent SBW25 cells become rare on the aspen primary root surface 30 days after inoculation of the RMI-Chip. SBW25 cells preferentially occupy the space between root epidermal cells.

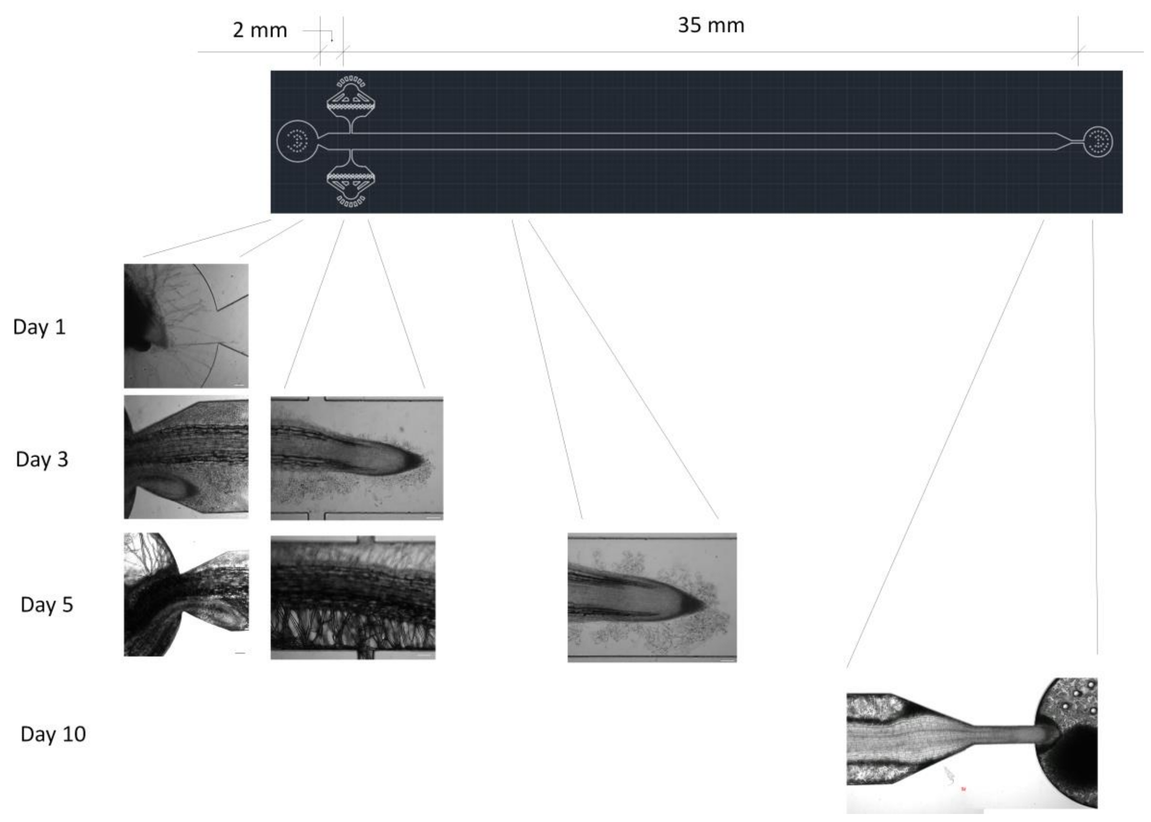

Supplementary Figure S10. Example of an aspen root growing in the RMI-chip. Root growth was monitored in one of the channels of the RMI-Chip using a 1x objective which allows us to cover the length of the chamber in 6 frames. For the first 2 days, this root grew slowly (~ 1.6 mm /day) out of the nutrient flow until it reached the side channels where the flow enters the chamber. Then, root growth accelerated to ~ 4.7 mm / day, making it the fastest growing root we observed with our setup. At day 10, the root had reached the end of the chamber and blocked the exhaust channel. More generally, we observed that in our system root growth varied substantially between seedlings and from experiment to experiment.

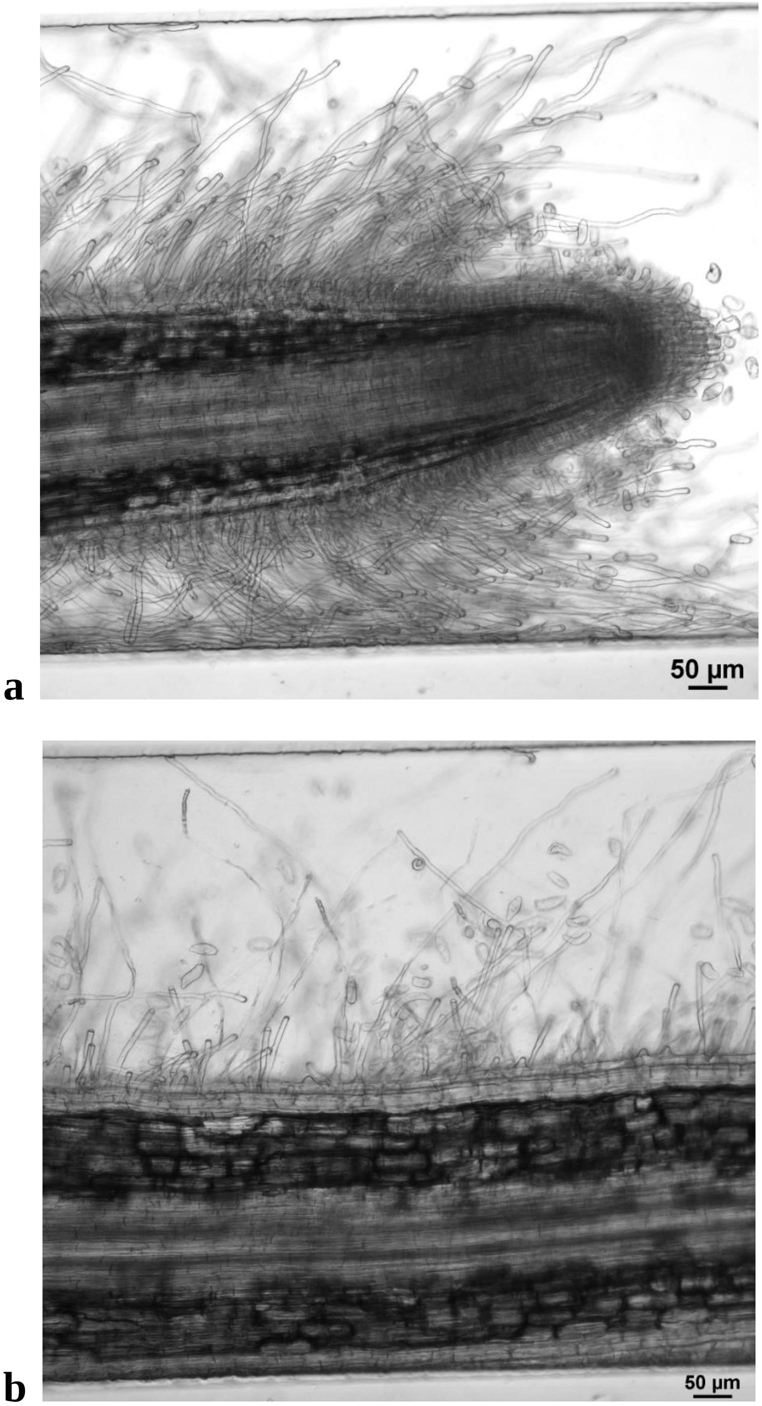

Supplementary Figure S11. Lack of colonization of rice root with fluorescently labeled *P. fluorescens*. *P. fluorescens* SBW25 strains expressing dsRed or mNeonGreen were co-inoculated on rice roots growing in the RMI-chip. After incubation under stopped flow, the flow was restored, and the root was imaged a day later. As opposed to the aspen root, no microbes attached to the surface of

the root tip (A) or mid-section (B) could be detected. This experiment was replicated 3 times independently.

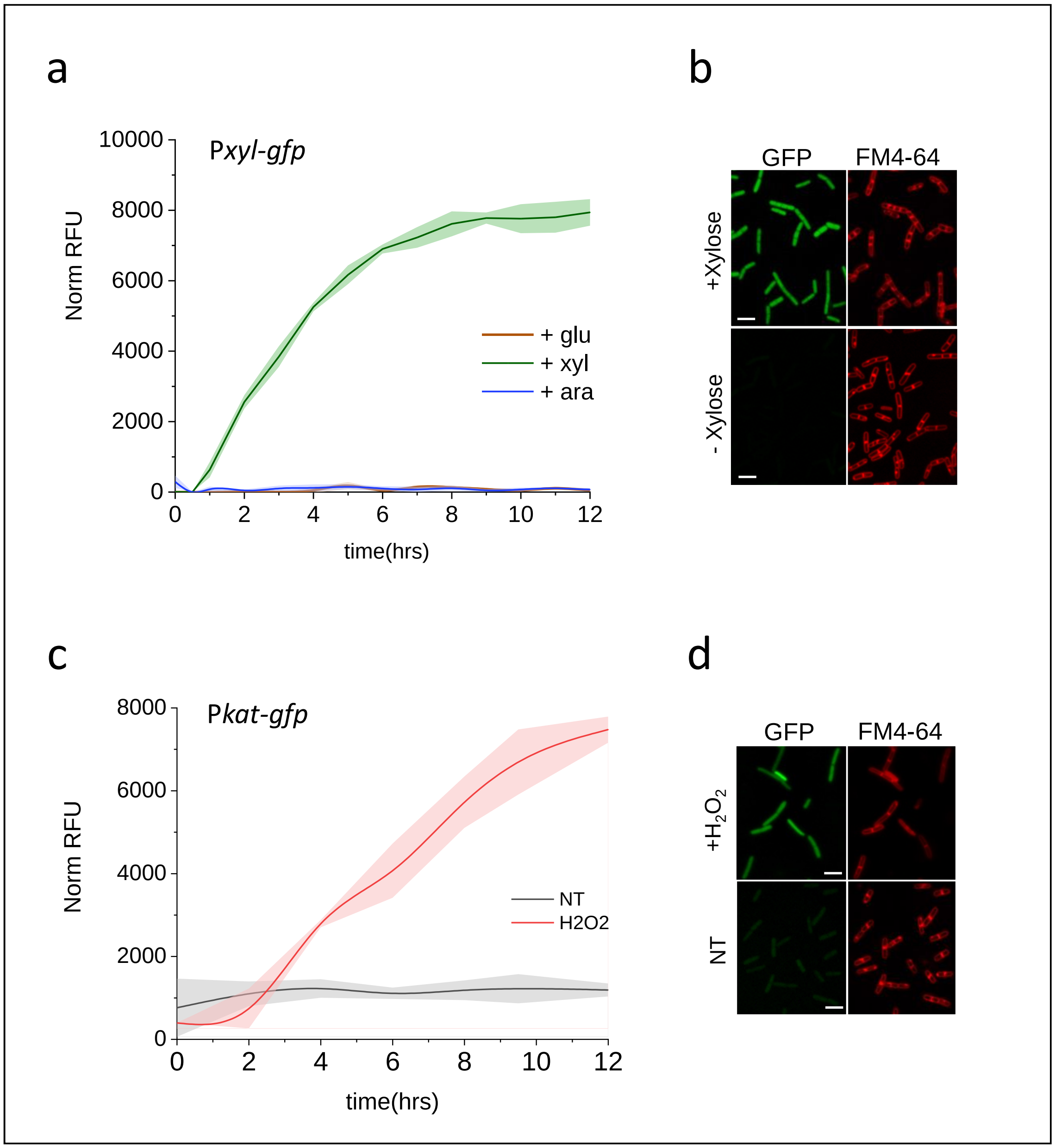

Supplementary Figure S12. **Characterization of *Bacillus subtilis* biosensor strains**. (a) For the xylose biosensor strain, GFP expression and cell density were measured in the presence of xylose, glucose or arabinose. Normalized RFU values were calculated after background subtraction (see Methods). Values correspond to the average of 4 independent measurements. Standard deviations are represented as filled areas. (b) At the 5-hour time point a small aliquot of cells were stained with the red membrane stain FM4-64 and imaged on a confocal microscope. GFP expression in the presence of xylose appears homogeneous in the cell population. (c) ROS biosensor cells were incubated in the presence or absence of 0.015% H_2O_2. Normalized RFU values were calculated after background subtraction. The average values of 4 independent experiments are plotted. Standard deviations are represented as filled areas. (d) At the 5-hour time point a small aliquot of cells were stained with the red membrane stain FM4-64 and imaged on a confocal microscope, revealing a low basal expression of GFP in absence of H_2O_2 and upon H_2O_2 treatment, a substantial level of heterogeneity in the cell population. Scale bar 5μm.

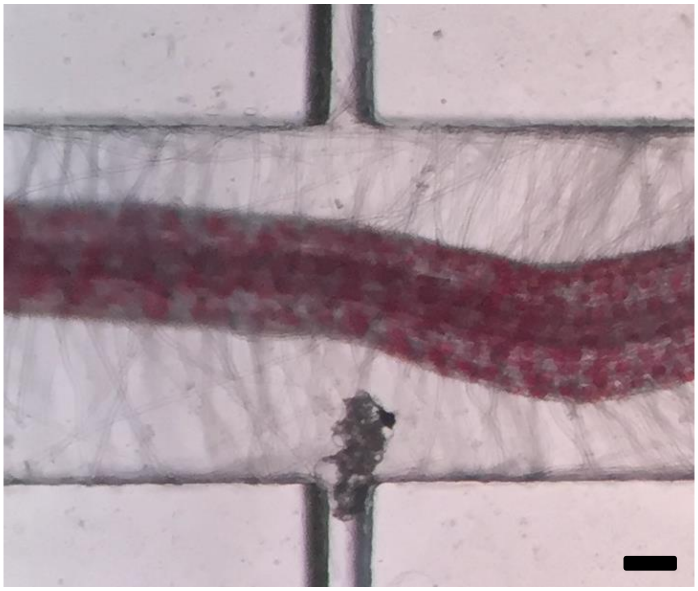

a

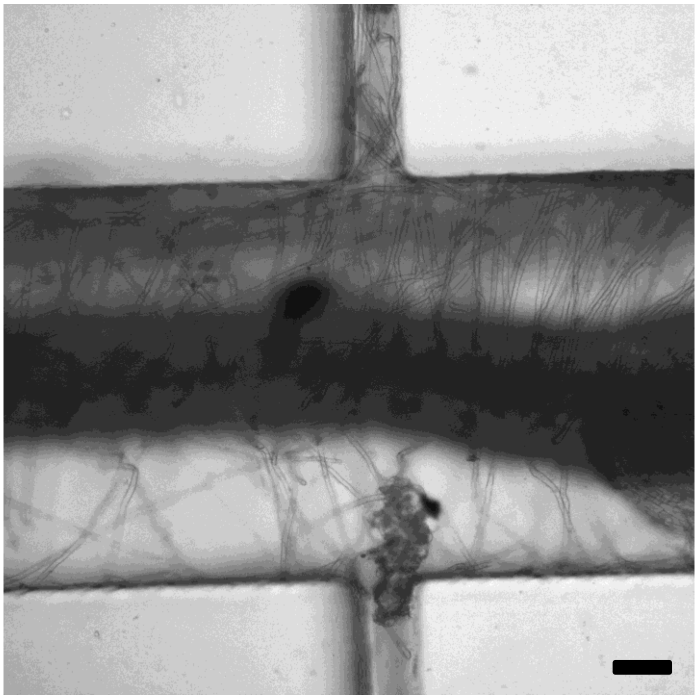

b

Supplementary Figure S13. **Imaging root growth in a 400 µm- deep RMI-chip chamber**. RMI-chip with a 400 µm channel depth enables root growth but does not position the root close enough from the glass coverslip, making it suboptimal for microscopic observations using objectives with standard working distances. (a) Root growing in a 400 µm-deep channel imaged with a regular color camera mounted on an inverted microscope. The red color is due to anthocyanin pigments produced by the root cells. Focus on the root surface is lost in the left part of the image. (b) With our confocal microscope setup, the root surface cannot be brought into focus at this location. Scale bar 100 µm.

S1

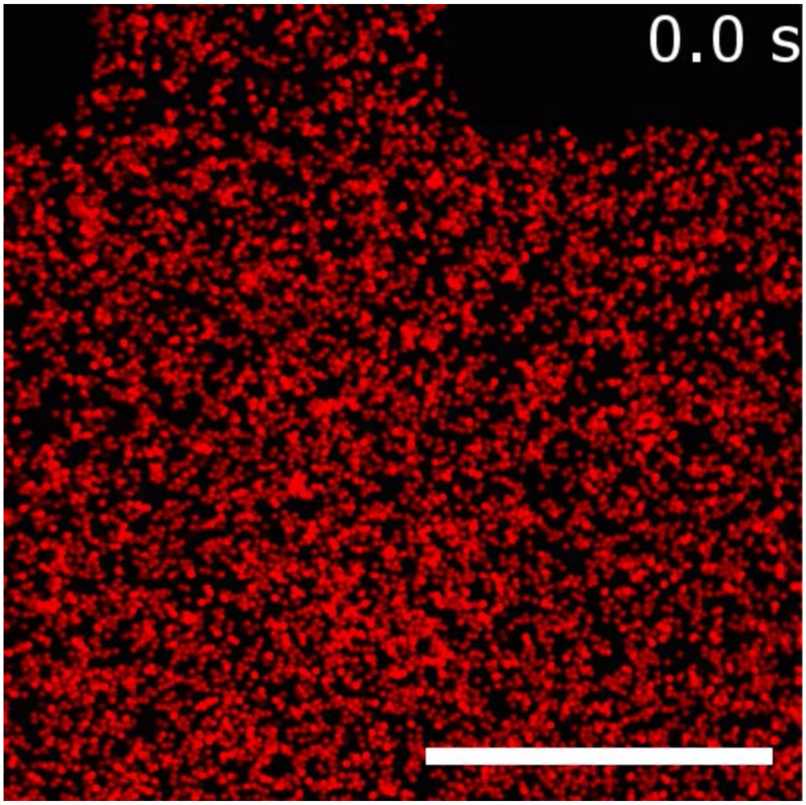

S2

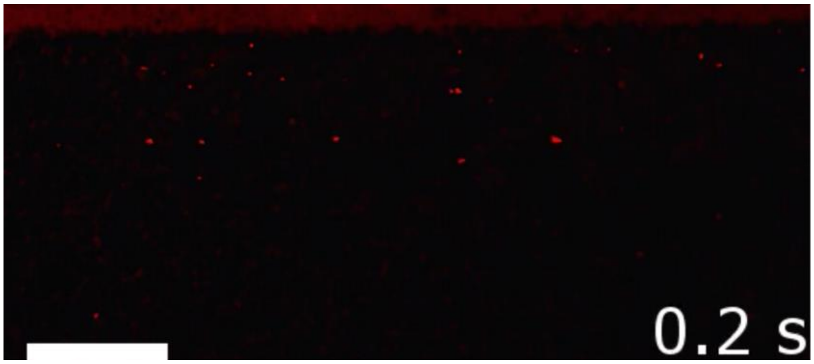

Video S1 and S2. Flow characteristics of the 400 µm x 800 channel section of the RMI-Chip. The flow characteristics in the channel were measured with fluorescently labelled styrene beads. Without perfusion, slight bulk material movement was detected (S1). At 1 µL/min, laminar flow was observed with a measured 50 µm/s bead velocity (S2; bar = 100 µm).

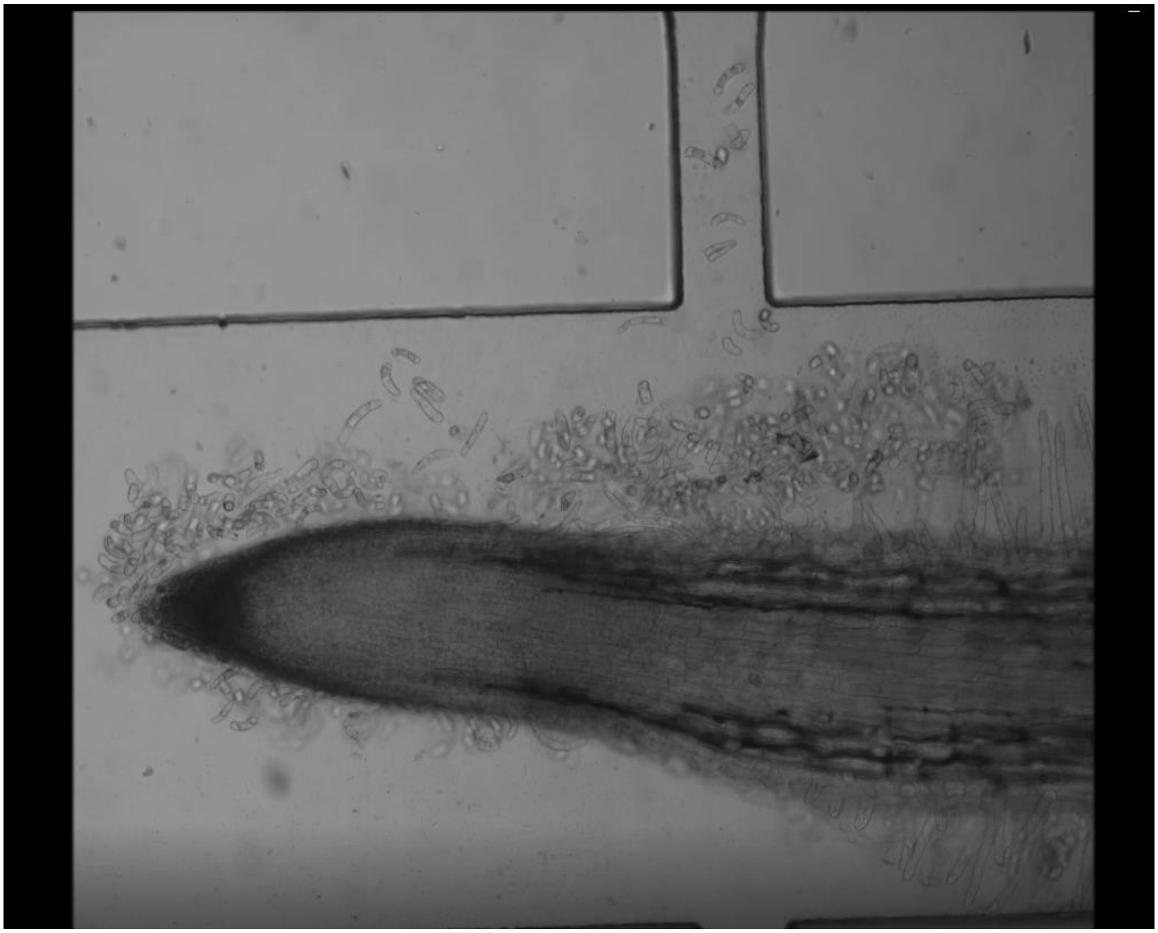

Video S3. Flow of nutrient into the RMI-Chip at 0.02 µL/min. The minimal flow rate of nutrient solution that did not affect root growth, preserved root morphology, including root cap, root hairs and mucilage, while keeping out air bubbles. The nutrient is supplied from the channel on top in this video.

Table S1. Oligonucleotides, plasmids and strains.

Oligonucleotides

Name	Target	Enzymes	Features	Sequence
MFN121	GFPsp-Fw	Spe1	RBS	GCAT*ACTAGT*AGG AGG ACA AAC ATG GTT TCT AAA GGT GAA G
MFN123	GFPsp-Rv	Pst1	2 stop codons	GCAA*CTGCAG*TG CTC ATT ATT ATT TAT ACA ATT CAT CCA TAC CAT GTG T
MFN63	pXyl-Fw	EcoR1		AGGATGATTTCT*GGAATT*CGCGG
MFN64	pXyl-Rv	Spe1		GACTGCAGCGGCCGCT*ACTAGT*A

Strains

Strain	bacteria	Background	source*	genotype
3a1	B. subtilis	NCIB 3610	BGSC	wild type
MMB1023*	B. subtilis	NCIB 3610	Babic *et al.*, 2011	
3a1-mCherry**	B. subtilis	NCIB 3610		cgeD::[(Ppen-mCherry)]kn
1A976	B. subtilis	SKC6	BGSC	Em^R his nprE18 aprE3 eglSΔ102 bglT/bglSΔEV lacA::PxylA-comK
1A1010*	B. subtilis	SH517	BGSC	Spc^R pheA1 trpC2
BSBIOA1	B. subtilis	SKC6	this work	Em^R his nprE18 aprE3 eglSΔ102 bglT/bglSΔEV lacA::PxylA-comK amyE::[Pxyl-GFP]cat
BSBIOA2	B. subtilis	SKC6	this work	Em^R his nprE18 aprE3 eglSΔ102 bglT/bglSΔEV lacA::PxylA-comK amyE::[PkatA-gfp] spc
BSBIOA4	B. subtilis	NCIB 3610	this work	cgeD::[(Ppen-mCherry)]kn amyE::[Pxyl-GFP]cat
BSBIOA5	B. subtilis	NCIB 3610	this work	cgeD::[(Ppen-mCherry)]kn amyE::[PkatA-gfp] spc
SBW25_neon	P. fluorescens	SBW25	Wilton *et al.*, 2017	wild type with plasmid pSW002-Pc-mNeonGreen
SBW25_red	P. fluorescens	SBW25	Wilton *et al.*, 2017	wild type with plasmid pSW002-Pc-DsRed-Express2

* donor; ** recipient; BGSC http://www.bgsc.org
